# GPR81 Regulates MCT1 Membrane Translocation Through a PKA-Dependent Signaling Pathway in Rat Podocytes

**DOI:** 10.3390/ijms27177563

**Published:** 2026-08-24

**Authors:** Klaudia Grochowalska, Maria Szrejder, Irena Audzeyenka, Agnieszka Piwkowska

**Affiliations:** Laboratory of Molecular and Cellular Nephrology, Mossakowski Medical Research Institute, Polish Academy of Sciences, 02-106 Warsaw, Poland; mszrejder@imdik.pan.pl (M.S.); iaudzeyenka@imdik.pan.pl (I.A.)

**Keywords:** MCT1, GPR81, PKA, H_2_O_2_, endocytosis, podocyte

## Abstract

Podocytes and their foot processes form a functional layer of the glomerular filtration barrier. Due to their unique morphology and function, podocytes employ distinct nutrient pathways to maintain the bioenergetic balance, with lactate being one of several available energy substrates. Enhanced lactate intake modulates the redox state of the cell by increasing mitochondrial respiration and reactive oxygen species production. Monocarboxylate transporter 1 (MCT1) is the primary lactate transporter, and alterations in its surface expression may contribute to the regulation of lactate uptake in podocytes. Beyond its metabolic role, lactate also acts as a crucial signaling molecule by binding to G-protein-coupled receptor 81 (GPR81), mediating a wide range of physiological effects through the inhibition of protein kinase A (PKA). The present study investigated novel regulatory mechanisms of MCT1 internalization, which depend on GPR81 signaling and PKA activity in primary rat podocytes, through the biotinylation assay. Surprisingly, both PKA inhibition (with H89 and PKI 14–22) and activation (with 8-bromo-cAMP and H_2_O_2_) increased MCT1 internalization. GPR81 was also found to regulate MCT1 membrane trafficking, likely through the modulation of PKA activity but also potentially through PKA-independent mechanism. Additionally, general dynamic changes in endocytic activity were detected under the present conditions with pHrodo-dextran fluorescence analysis. These results suggest that the modulation of PKA activity and GPR81 signaling may regulate lactate transport via MCT1, thereby ensuring its proper metabolic function in podocytes.

## 1. Introduction

Lactate was once regarded as merely a byproduct of anaerobic metabolism, but it is now recognized as a key regulator of metabolic homeostasis, even under aerobic conditions. It serves as a major fuel source for the tricarboxylic (TCA) acid cycle, a precursor for gluconeogenesis, and a signaling molecule that is capable of modulating various physiological processes [1]. The rapid transport of lactic acid across the plasma membrane is fundamental for cellular metabolism and intracellular pH homeostasis. This transport is facilitated by proton-linked monocarboxylate transporters (MCTs) [2], among which MCT1 is the best characterized and plays a major role in L-lactate uptake for oxidation [3,4,5].

Podocytes are highly differentiated epithelial cells that form the glomerular filtration barrier. They exhibit significant metabolic demands and require precise metabolic regulation to sustain their function [6]. Given their high energy requirements, lactate serves as an important metabolic substrate that can be incorporated into the TCA cycle within mitochondria. Our recent findings demonstrated that podocytes actively utilize lactate for energy production and possess a well-developed lactate homeostasis system, including a specialized lactate transport mechanism [7]. The presence of MCTs in podocytes has been confirmed, with rapid alterations in their expression levels in response to high extracellular lactate concentrations and glucose deprivation [7]. Notably, the mitochondrial localization of MCT1 and MCT2 under these conditions was identified [8], suggesting a significant shift toward oxidative phosphorylation and reinforcing the role of lactate as a key regulator of mitochondrial metabolism [9,10]. As a result of greater lactate oxidation, an increase in the production of reactive oxygen species (ROS) should be expected [11]. Lactate has also been shown to generate H_2_O_2_ independently of the respiratory chain through the activity of L-lactate oxidase [12]. This dual role of lactate—as both a substrate for mitochondrial metabolism and a redox signaling molecule—highlights its essential role in podocyte energy metabolism [13,14,15].

Beyond its metabolic function, lactate also acts as a potent signaling molecule that is involved in cellular communication, metabolic regulation, gene expression, and immune responses. Notably, lactate serves as a ligand for G-protein-coupled receptor 81 (GPR81), also known as hydroxycarboxylic acid receptor 1 (HCAR1) [16]. Our recent study revealed the impact of lactate signaling through GPR81 on lipolysis in podocytes, further underscoring its functional significance in podocyte metabolism [17].

This dual metabolic and signaling role of lactate requires tightly regulated mechanisms for its transport and homeostasis. However, despite extensive research on MCT-mediated lactate transport, the precise regulatory mechanisms that govern its trafficking remain largely unexplored, particularly in podocytes.

In skeletal muscle, MCT1 regulation has been linked to protein kinase C (PKC)- and protein kinase A (PKA)-mediated pathways [3]. A previous study by Smith et al. [18] demonstrated that cyclic adenosine monophosphate (cAMP) induces MCT1 dephosphorylation and its internalization into caveolae and early endosomes in the RBE4 rat brain cerebrovascular endothelial cell line, emphasizing the role of vesicular trafficking in controlling MCT1 surface expression and function. In this study, we investigated the PKA-dependent internalization of MCT1 in primary rat podocytes, since PKA acts as the direct downstream effector of GPR81.

Previous findings indicated that cell surface transporters undergo active turnover via the endosomal–lysosomal sorting system [19]. Endocytosis, an energy-dependent process, is responsible for internalizing membrane proteins and directing them to intracellular compartments, including lysosomes, trans-Golgi network, endoplasmic reticulum, and Rab11-positive recycling endosomes. Both clathrin- and caveolin-dependent endocytic pathways regulate protein localization and transport, with caveolae-mediated trafficking being particularly well characterized in capillary endothelial cells [20]. The involvement of caveolin-1, a key caveolae component, has been demonstrated through its interaction with podocyte slit diaphragm proteins, such as nephrin and CD2-associated protein (CD2AP), suggesting a potential role for endocytosis in modulating glomerular hemodynamics in response to metabolic changes [21]. Rab guanosine triphosphatases, particularly Rab5, act as pivotal regulators of vesicular trafficking by orchestrating vesicle budding, docking, fusion, and cargo sorting in early endosomes. Membrane trafficking integrates protein networks with signaling cascades, serving as a vital communication system between the extracellular environment and intracellular compartments [22]. In podocytes, the endocytic pathway plays a crucial role in maintaining cellular homeostasis by regulating surface receptor recycling, retrieving filtered proteins, such as albumin, for degradation, and modulating intracellular signaling pathways [23,24,25,26]. Additionally, the efficient trafficking and regulation of metabolic surface transporters may allow adaptation to changing metabolic demands, ensuring optimal nutrient exchange, energy balance, and functional stability.

Given that lactate is an important metabolic substrate for podocytes, the rapid and dynamic trafficking of MCT1 between cellular compartments is likely crucial for adaptation to changes in the microenvironment. Lactate transport regulated by the GPR81-mediated modulation of intracellular cAMP levels may play a central role in maintaining podocyte homeostasis. Additionally, ROS, such as H_2_O_2_, can modulate PKA activity independently of cAMP. Thus, the present study investigated the impact of GPR81 activation and cAMP-dependent and -independent modulation of PKA activity on MCT1 internalization. Our aim was to uncover molecular mechanisms that govern MCT1 turnover in podocytes.

## 2. Results

### 2.1. Modulation of PKA Activity Induces MCT1 Internalization and Is Associated with Rab5a- and Caveolin-1-Dependent Endocytosis

**Figure 1:**
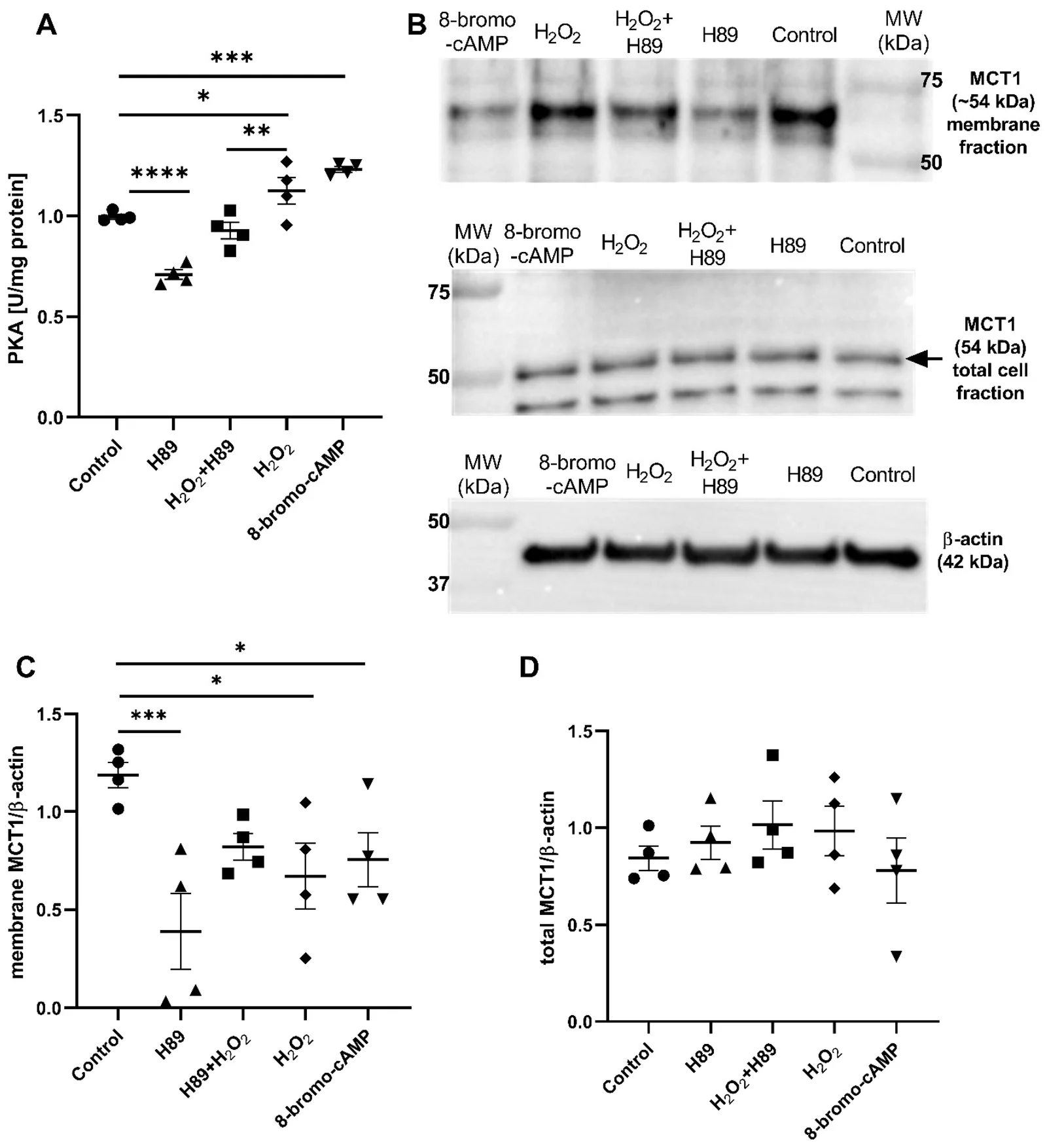
Alteration of PKA activity affects MCT1 membrane localization in primary rat podocytes. (**A**) PKA activity (U/mg of protein). (**B**) Immunoblotting membranes showing biotinylated (membrane) and total (whole cell lysate) fractions of MCT1 in podocytes. (**C**) Membrane content of MCT1 and (**D**) total amount of MCT1 protein in cell lysates. Cells were treated with: 8-bromo-cAMP (100 µM, 5 min), H_2_O_2_ (100 µM, 5 min), H89 (10 µM, 1 h), or H_2_O_2_ + H89. * *p* < 0.05, ** *p* < 0.01, *** *p* < 0.001, **** *p* < 0.0001, one-way ANOVA, Sidak’s multiple comparisons, *n* = 4.

MCT1 facilitates the bidirectional transport of lactate across the plasma membrane [27], and its endocytosis is regulated by multiple signaling pathways [18,19]. Additionally, MCT1 function is regulated by β-adrenergic receptor signaling via the cAMP–PKA pathway [28]. To investigate the mechanism of MCT1 regulation in podocytes, first the impact of PKA activity modulation on MCT1 protein levels was assessed. The cAMP analog 8-Br-cAMP was used as specific PKA activator, and H89 as a PKA inhibitor. Additionally, PKA can be activated in a cAMP-independent manner through ROS (e.g., H_2_O_2_) and PKA dimerization [20]. After 8-bromo-cAMP (100 µM, 5 min) and H_2_O_2_ (100 µM, 5 min) treatments PKA activity was increased by 24% (1.23 ± 0.014; *p* = 0.0005) and 13% (1.13 ± 0.066; *p* = 0.028), respectively (Figure 1A). Exposure to H89 (10 µM, 1 h) decreased PKA activity by 29% (0.709 ± 0.024; *p* < 0.0001) compared to control (0.996 ± 0.012) (Figure 1A). Combined treatment with H_2_O_2_ and H89 (H_2_O_2_ + H89) resulted in reduced PKA activity by 7% (0.927 ± 0.042; *p* = 0.002) compared with H_2_O_2_ alone (Figure 1A).

The next step was to determine whether PKA activity modulation had an effect on MCT1 protein levels. Both inhibitor and activator of PKA activity decreased MCT1 protein levels in the cell membrane, while its total cellular amount remained unchanged (Figure 1B–D). MCT1 protein levels in the cell membrane decreased by 67% after incubation with H89 (0.388 ± 0.192; *p* = 0.0009) and decreased by 43% after 8-bromo-cAMP incubation (0.754 ± 0.138; *p* = 0.041) (Figure 1C). Membrane levels of MCT1 were also diminished by 38% after H_2_O_2_ treatment (0.670 ± 0.169; *p* = 0.01) compared to control (1.187 ± 0.065) (Figure 1C). Combined exposure of podocytes to H_2_O_2_ + H89 abolished the effects of individual agents on MCT1 levels (Figure 1C).

**Figure 2:**
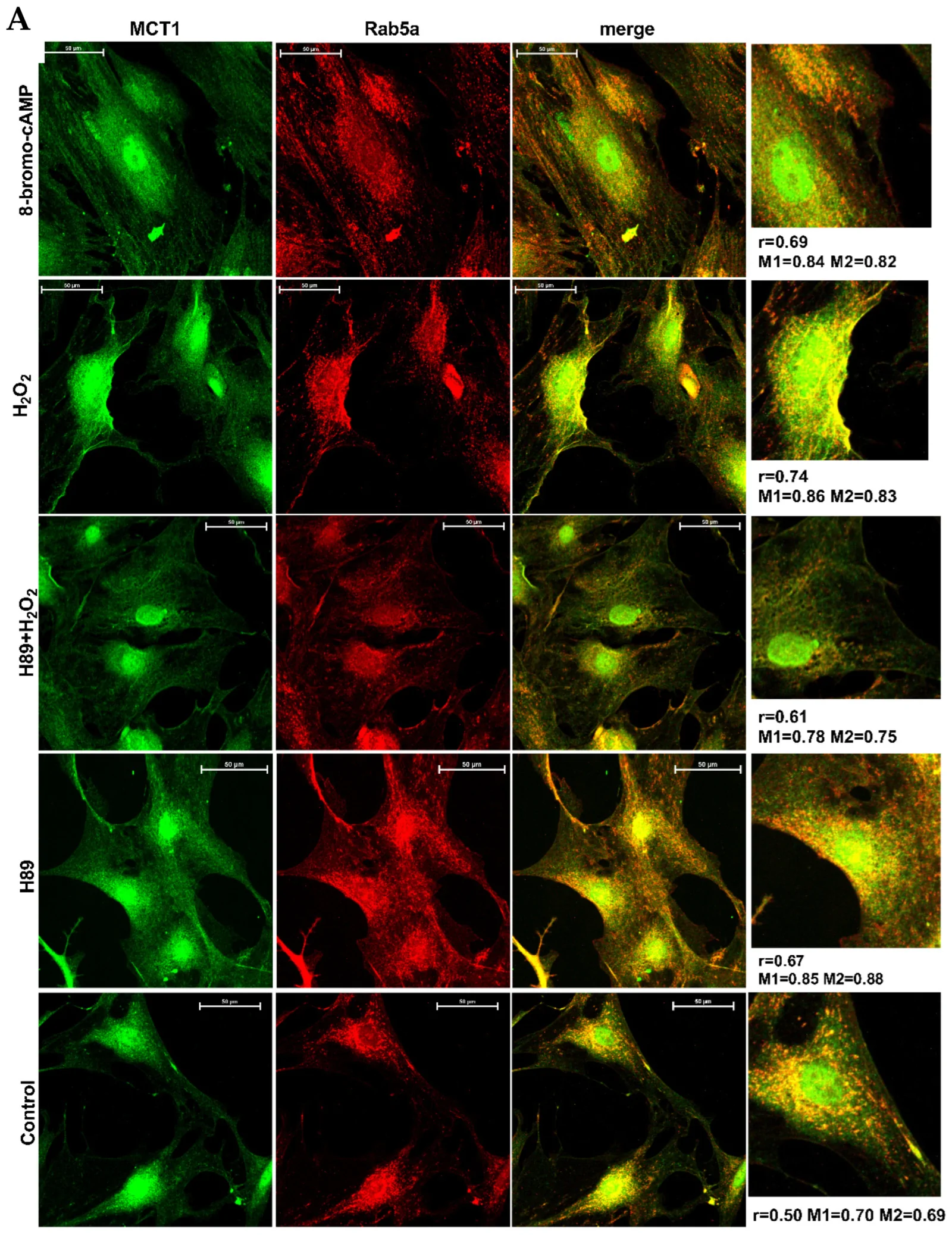

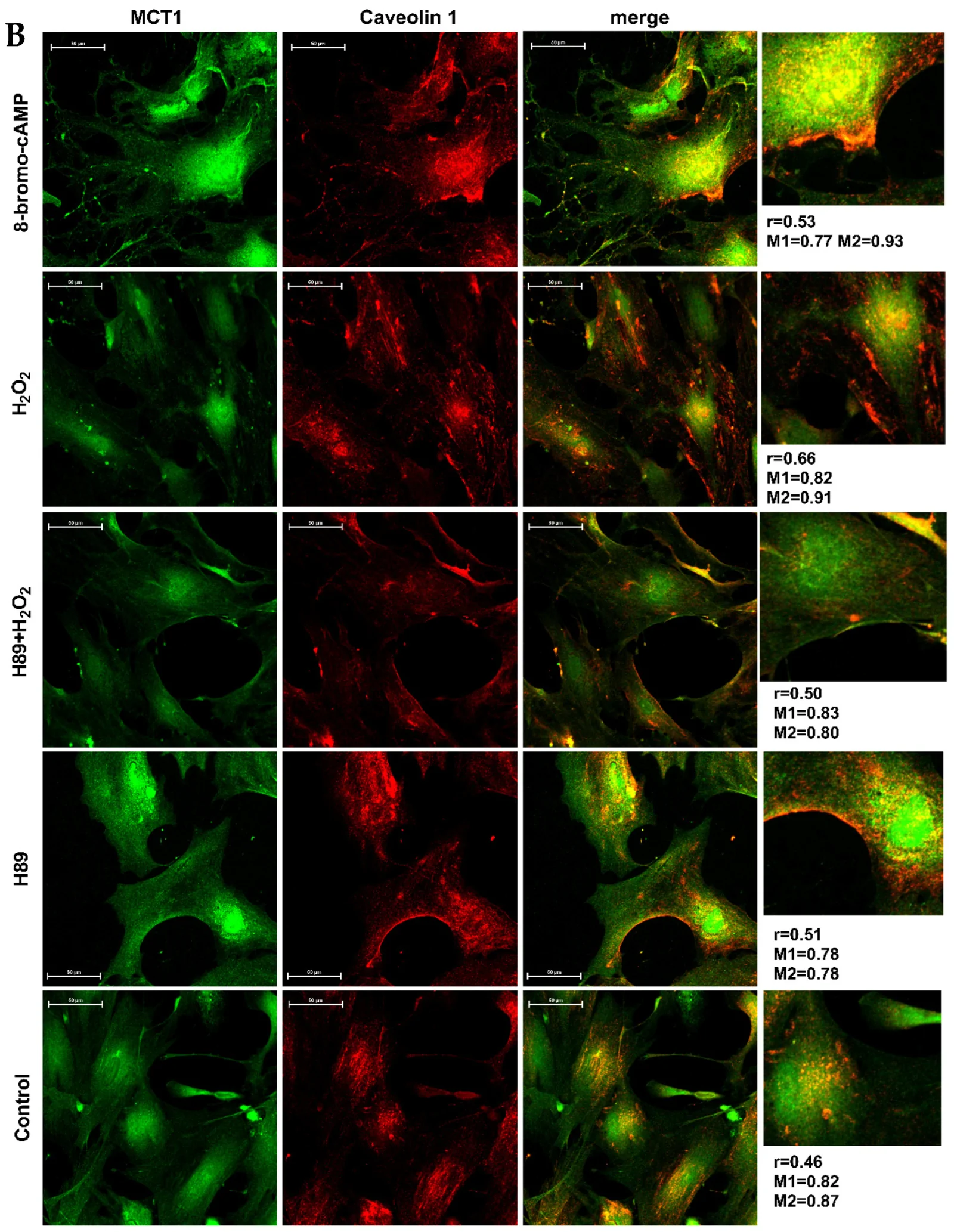
Changes in PKA activity affect caveolin-dependent membrane translocation of MCT1 in podocytes. (**A**) Immunofluorescent co-staining of MCT1 and Rab5a in podocytes. (**B**) Immunofluorescent staining of MCT1 and caveolin-1 in podocytes. r—Pearson’s correlation coefficient; M1, M2—Manders’ overlap coefficients. Cells were treated with: 8-bromo-cAMP (100 µM, 5 min), H_2_O_2_ (100 µM, 5 min), H89 (10 µM, 1 h), or H_2_O_2_ + H89. Podocytes were visualized using re-scan confocal microscopy (Nikon Eclipse Ti confocal microscope, Nikon Corporation, Tokyo, Japan). Scale bar is 50 µm.

Previous studies reported a cAMP-dependent increase in MCT1 endocytosis into caveolae via a clathrin-independent pathway in rat brain endothelial cells [18]. Therefore, we investigated whether changes in MCT1 membrane level, observed upon PKA activity modulation, involve caveolin-1- and Rab5a-dependent pathways. Immunofluorescence analyses of MCT1 and Rab5a (Figure 2A) and MCT1 and caveolin-1 (Figure 2B) co-staining were performed. Treatment with either H89 or H_2_O_2_ alone increased the Rab5a and MCT1 immunostaining intensity near the cell membrane. In cells that were treated with both H89 and H_2_O_2_, the signal was similar to control. Interestingly, cells that were treated with 8-bromo-cAMP showed the abundant expression of MCT1 both in cytoplasm and near the cell membrane and immunostaining intensity of Rab5a was more concentrated near the plasma membrane than in its central part (Figure 2A). Moreover, quantitative colocalization analysis revealed an increased linear correlation between MCT1 and Rab5a after H_2_O_2_ treatment by 47% (r = 0.74, *p* = 0.01) and 8-bromo-cAMP treatment by 38% (r = 0.69, *p* = 0.04) compared to control (r = 0.50) (Figure S1A). These results were accompanied by a statistically significant increase in Manders’ coefficient M_1_ (Rab5a in MCT1-positive regions) following treatment H_2_O_2_ by ~23% (M_1_ = 0.86, *p* = 0.003) and 8-bromo-cAMP by ~20% (M_1_ = 0.84, *p* = 0.04) compared to control (M_1_ = 0.70) (Figure S1C). In contrast, no statistically significant changes were observed for M_2_ (MCT1 in Rab5a-positive regions) (Figure S1D). Treatment with H89 increased Manders’ coefficient M_1_ by ~21% (0.85, *p* = 0.03) and M_2_ by ~28% (0.88, *p* = 0.01) compared to control (M_1_ = 0.70; M_2_ = 0.69) (Figure S1C,D).

Immunofluorescent co-staining of MCT1 and caveolin-1 showed that H89 treatment intensified the signal of caveolin-1 within the cytoplasm and near the cell membrane, whereas the simultaneous use of H89 and H_2_O_2_ (H89 + H_2_O_2_) mitigated this effect (Figure 2B). PKA activation via 8-bromo-cAMP exhibited enhanced fluorescent signal from caveolin-1 near the cell membrane, whereas activation via H_2_O_2_ exhibited elevated membrane immunostaining of caveolin-1 and accumulation of MCT1-corresponding signal in punctate pattern at the cell periphery, although without evident colocalization with caveolin-1 (Figure 2B). No statistically significant changes were observed in the colocalization of MCT1 and caveolin-1 (Figure S1B,E,F), suggesting local alterations in fluorescence intensity of caveolin-1.

**Figure 3:**
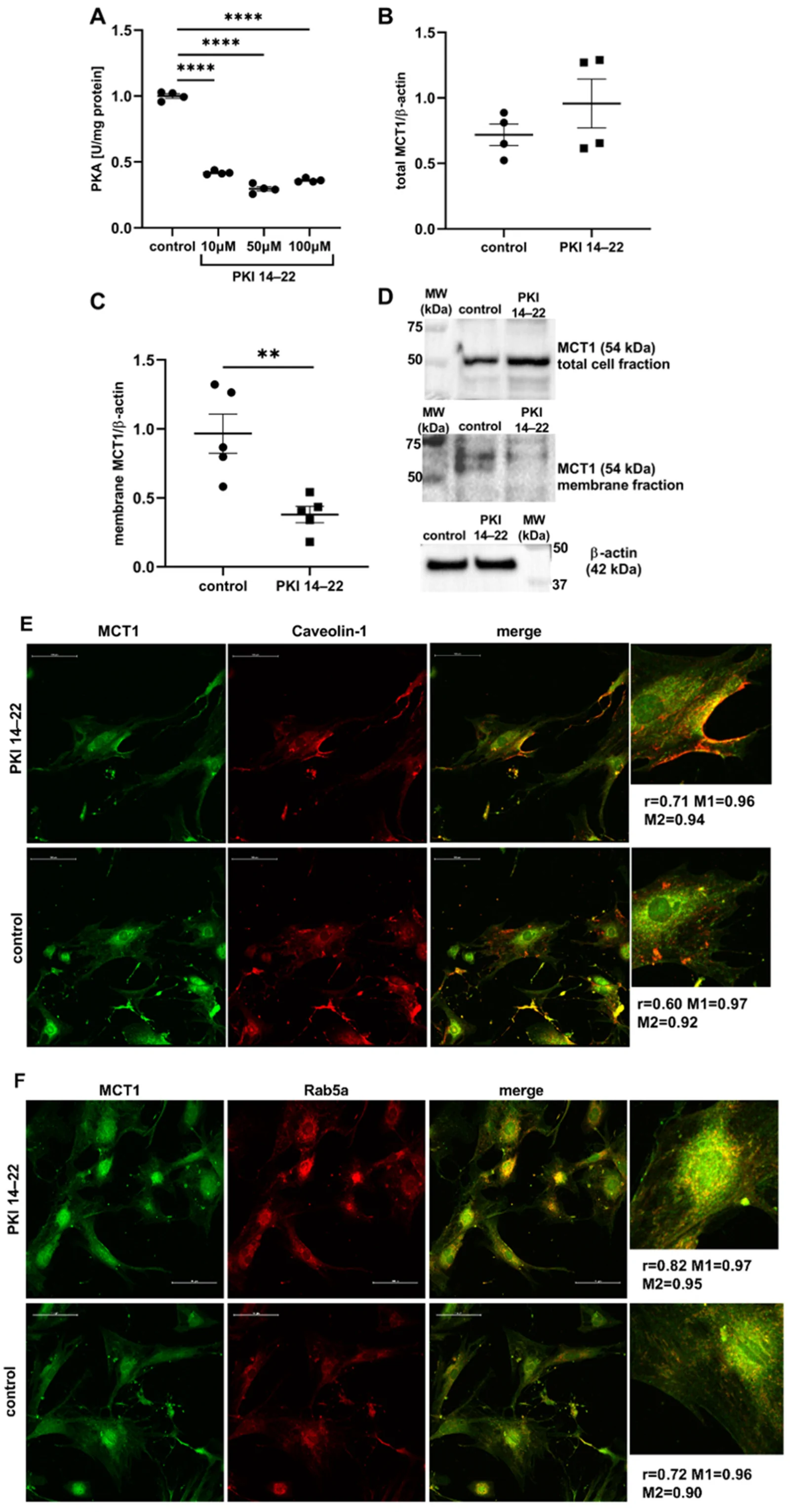
A competitive substrate-site PKA inhibitor, PKI 14–22, increased caveolin-dependent internalization of MCT1. (**A**) PKA activity (U/mg of protein). (**B**) Total amount of MCT1 protein and (**C**) membrane content of MCT1. (**D**) Immunoblotting membranes showing biotinylated (membrane) and total (whole cell lysate) fractions of MCT1. (**E**) Immunofluorescent staining of MCT1 and caveolin-1 in podocytes. (**F**) Immunofluorescent staining of MCT1 and Rab5a in podocytes. r—Pearson’s correlation coefficient; M1, M2—Manders’ overlap coefficients. Cells were treated with PKI 14–22 (10 µM) for 30 min. Podocytes were visualized using re-scan confocal microscopy (Nikon Eclipse Ti confocal microscope, Nikon Corporation, Tokyo, Japan). Scale bar is 100 µm. ** *p* < 0.01, **** *p* < 0.0001, one-way ANOVA, Sidak’s multiple comparisons and unpaired *t*-test, *n* = 4–5.

To confirm these effects of PKA inhibition on MCT1 internalization, we conducted series of experiments using another specific PKA inhibitor—myristoylated PKI 14–22 amide. The activity of PKA was decreased under three different concentrations of PKI 14–22: 10 µM, 50 µM, and 100 µM, by 58%, 70%, 64% (1.86 ± 0.031; 1.32 ± 0.076; 1.60 ± 0.031; *p* < 0.0001), respectively, compared to control (4.44 ± 0.072) (Figure 3A). Based on these results, the lowest effective concentration of PKI 14–22 (10 µM) was chosen for the next experiments. Although PKI 14–22 did not have a statistically significant effect on the total amount of MCT1 protein in podocytes (Figure 3B,D), the membrane content of MCT1 was considerably decreased (by 61%; 0.380 ± 0.06 vs. 0.97 ± 0.142; *p* = 0.005) (Figure 3C,D). Immunofluorescent staining of MCT1, caveolin-1, and Rab5a under PKA-inhibiting conditions showed that PKI 14–22 treatment increased immunostaining intensity of both caveolin-1 and Rab5a proteins with MCT1 at the cell periphery (Figure 3E,F). Pearson’s coefficient indicated an increased overall spatial correlation between MCT1 and both caveolin-1 (r = 0.71, *p* = 0.03) by ~17% (Figure S2B), and Rab5a (r = 0.82, *p* = 0.04) by ~13% (Figure S2A) following PKI 14–22 treatment compared to control (r = 0.61, r = 0.72). No statistically significant changes were observed in the colocalization between MCT1 and both caveolin-1 and Rab5a (Figure S2C,D). Similar to H89, these changes in localization and linear intensity correlation of endocytosis markers (caveolin-1 and Rab5a) upon PKI 14–22 exposure may suggest altered endocytic dynamics in podocytes caused by PKA inhibition.

### 2.2. Activation of PKA Stimulates Cellular Endocytosis

**Figure 4:**
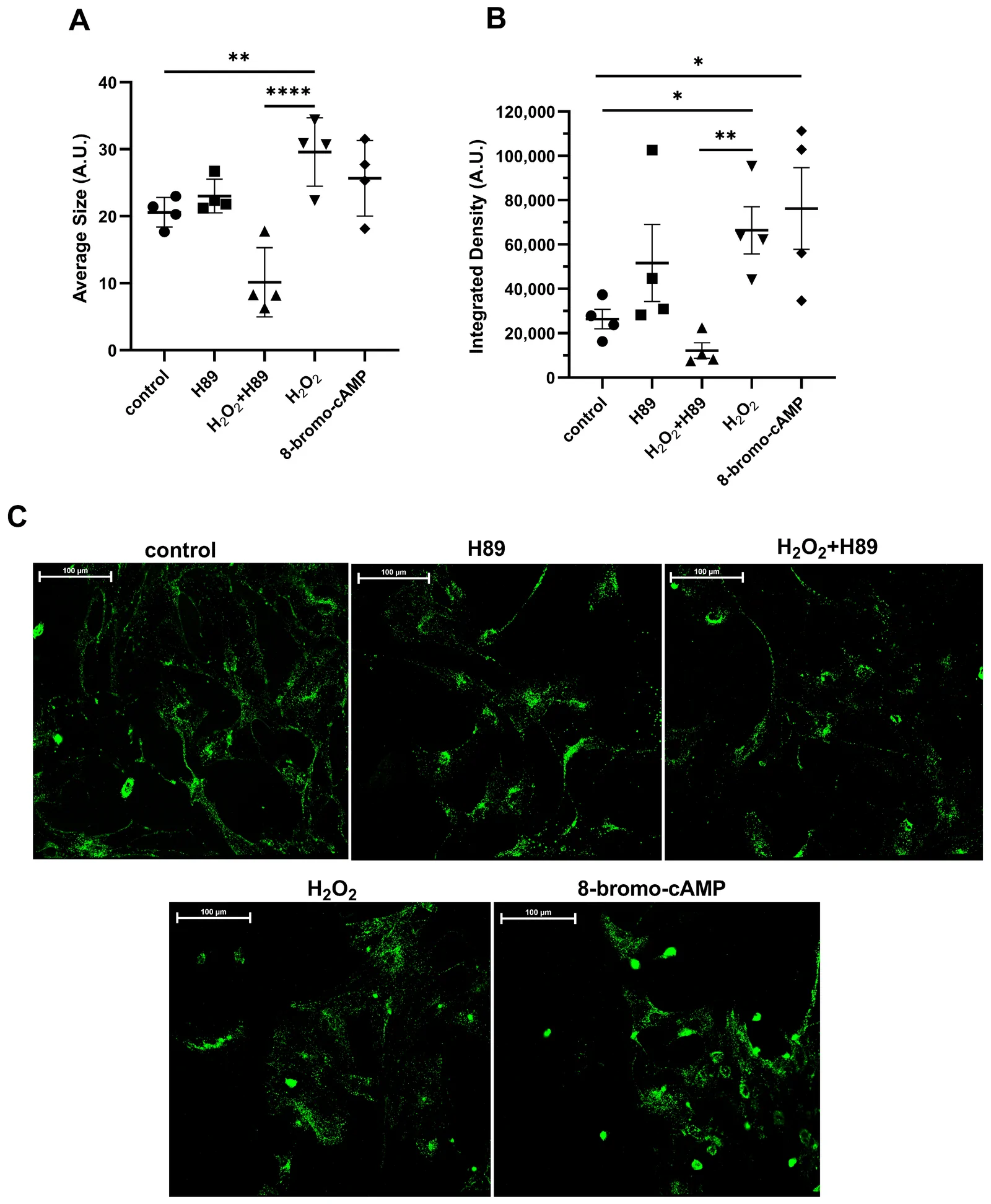
PKA activation stimulates cellular endocytosis in primary rat podocytes. (**A**) Average size and (**B**) integrated density of live early endosomes (pHrodo Green Dextran staining). (**C**) Intracellular distribution of early endosomes in podocytes. Cells were treated with: 8-bromo-cAMP (100 µM, 5 min), H_2_O_2_ (100 µM, 5 min), H89 (10 µm, 1 h), or H_2_O_2_ + H89. Cells were visualized using re-scan confocal microscopy (Nikon Eclipse Ti confocal microscope, Nikon Corporation, Tokyo, Japan). Scale bar is 100 µm. * *p* < 0.05, ** *p* < 0.01, **** *p* ≤ 0.0001, one-way ANOVA, Sidak’s multiple comparisons, *n* = 4.

The obtained results suggested that the potential relationship exists between PKA-dependent internalization of MCT1 and activation of caveolin-1-linked endocytosis. Therefore, our next studies aimed to investigate overall activity and intracellular localization of early endosomes in live podocytes following both the upregulation and inhibition of PKA activity (Figure 4). We observed an increase in the average size of early endosomes by 25% and 44% in the presence of 8-bromo-cAMP (25.7 ± 2.81; *p* = 0.121) and H_2_O_2_ (29.6 ± 2.56; *p* = 0.01)_,_ respectively, compared to control (20.6 ± 1.11) (Figure 4A). Moreover, podocyte treatment with 8-bromo-cAMP and H_2_O_2_ also increased the early endosome-corresponding fluorescence intensity by 227% and 153%, respectively (Figure 4B). PKA inhibitor, H89, did not alter the size or fluorescence intensity of early endosomes, but in cells exposed to H_2_O_2_ pre-treatment with H89 significantly reduced the stimulatory effect of H_2_O_2_ on endosomal size (by 65%) and on fluorescent intensity (by 81%; compared to the H_2_O_2_-treated cells) (Figure 4A,B). The intracellular distribution of endosomal vesicles was also affected by PKA modulators. Endosomes were localized in central areas of cells after 8-bromo-cAMP and H_2_O_2_ incubation with local clusters of endosomes near the cell membrane (Figure 4C). PKA inhibition with H89 and rescue treatment with both H_2_O_2_ and H89 shifted the localization of endosomal vesicles to more of a peripheral location, concentrated in foot processes of podocytes (Figure 4C).

### 2.3. MCT1 Internalization Might Be Regulated by Caveolin-1 Activity

**Figure 5:**
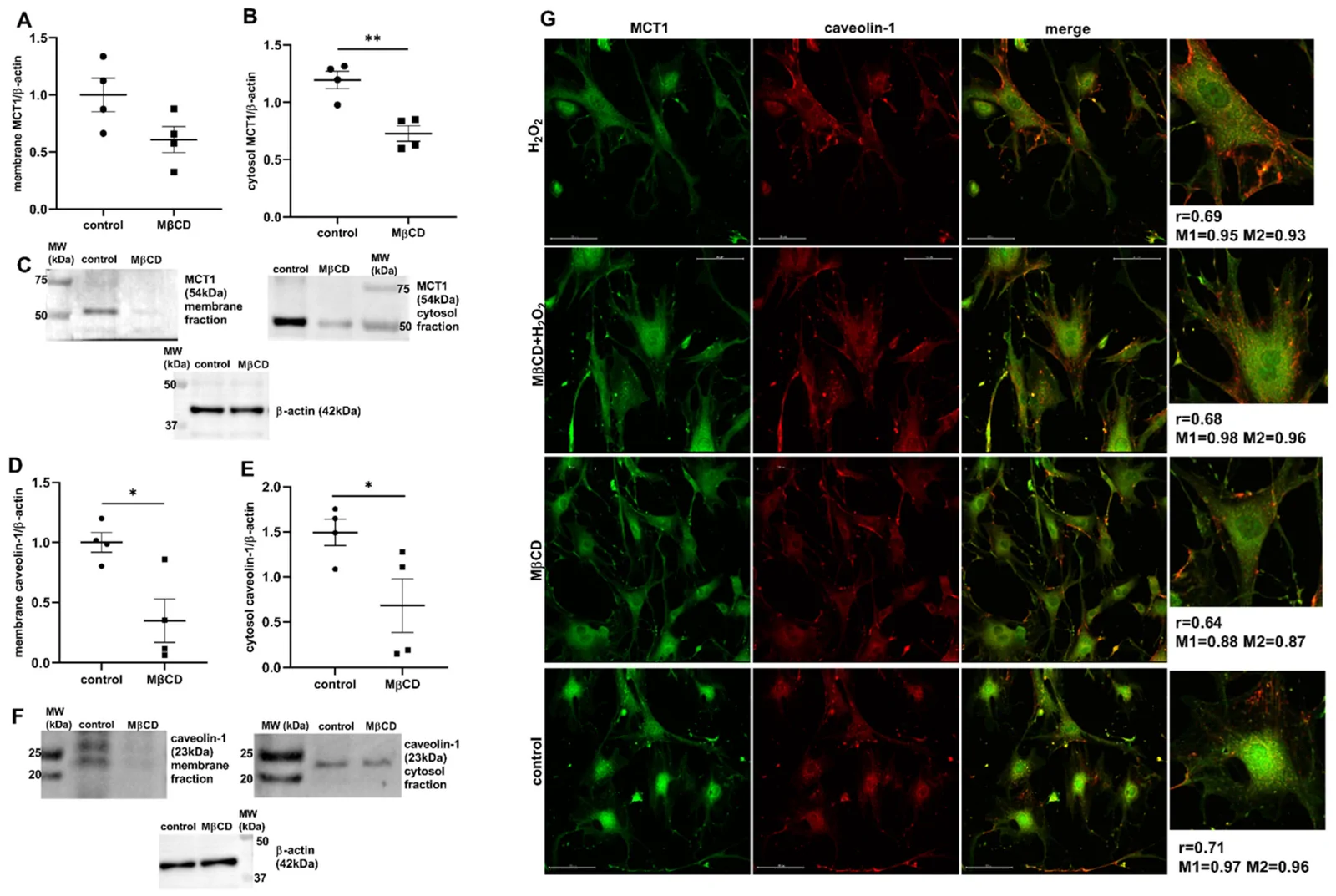
Endocytosis inhibitor (MβCD) decreased the level of MCT1 and caveolin-1 in podocytes. (**A**) MCT1 membrane content and (**B**) MCT1 cytosol levels. (**C**) Immunoblotting membranes showing MCT1 membrane and cytosolic fractions. (**D**) Caveolin-1 membrane and (**E**) caveolin-1 cytosol levels. (**F**) Immunoblotting membranes of caveolin-1 membrane and cytosol content. (**G**) Immunofluorescent staining of MCT1 and caveolin-1 in podocytes visualized using re-scan confocal microscopy (Nikon Eclipse Ti confocal microscope, Nikon Corporation, Tokyo, Japan). r—Pearson’s correlation coefficient; M1, M2—Manders’ overlap coefficients. Scale bar is 100 µm. * *p* < 0.05, ** *p* < 0.01, unpaired *t*-test, *n* = 4.

As far as we confirmed that both MCT1 membrane levels and caveolin-1-dependent endocytosis are related to changes in PKA activity, we decided to investigate whether MCT1 levels can be regulated by global modulation of cellular endocytic flux. We used MβCD to inhibit cholesterol-dependent endocytosis, including clathrin- and caveolin-mediated pathways (Supplementary Figure S3), and measured membrane and cytosolic content of MCT1. Both membrane (0.61 ± 0.11, *p* = 0.08) and cytosol (0.727 ± 0.067; *p* = 0.003) levels of MCT1 were decreased after MβCD treatment by 39% and by 14%, respectively, compared to controls (1 ± 0.15 and 1.19 ± 0.067) (Figure 5A–C), suggesting that endocytosis might be a potent regulatory pathway of MCT1 levels in podocytes and therefore can have a role in lactate transport. Moreover, MβCD treatment induced a significant change in cytosolic and membrane levels of caveolin-1, which were diminished by 45% (0.68 ± 0.298; *p* = 0.04) and 65% (0.347 ± 0.182; *p* = 0.02), respectively, compared to controls (1.49 ± 0.147, 1.00 ± 0.081) (Figure 5D–F).

The analysis of intracellular distribution of MCT1 and caveolin-1 in MβCD-treated podocytes revealed that the increased peripheral immunofluorescent intensity of caveolin-1 resulting from H_2_O_2_ treatment was abolished when cells were preincubated with MβCD (Figure 5G). MβCD treatment did not significantly change cellular localization of MCT1 and the colocalization of MCT1 and caveolin-1 (Figure S4).

### 2.4. H_2_O_2_ Exerts Pleiotropic Effect on Podocyte Endocytosis

**Figure 6:**
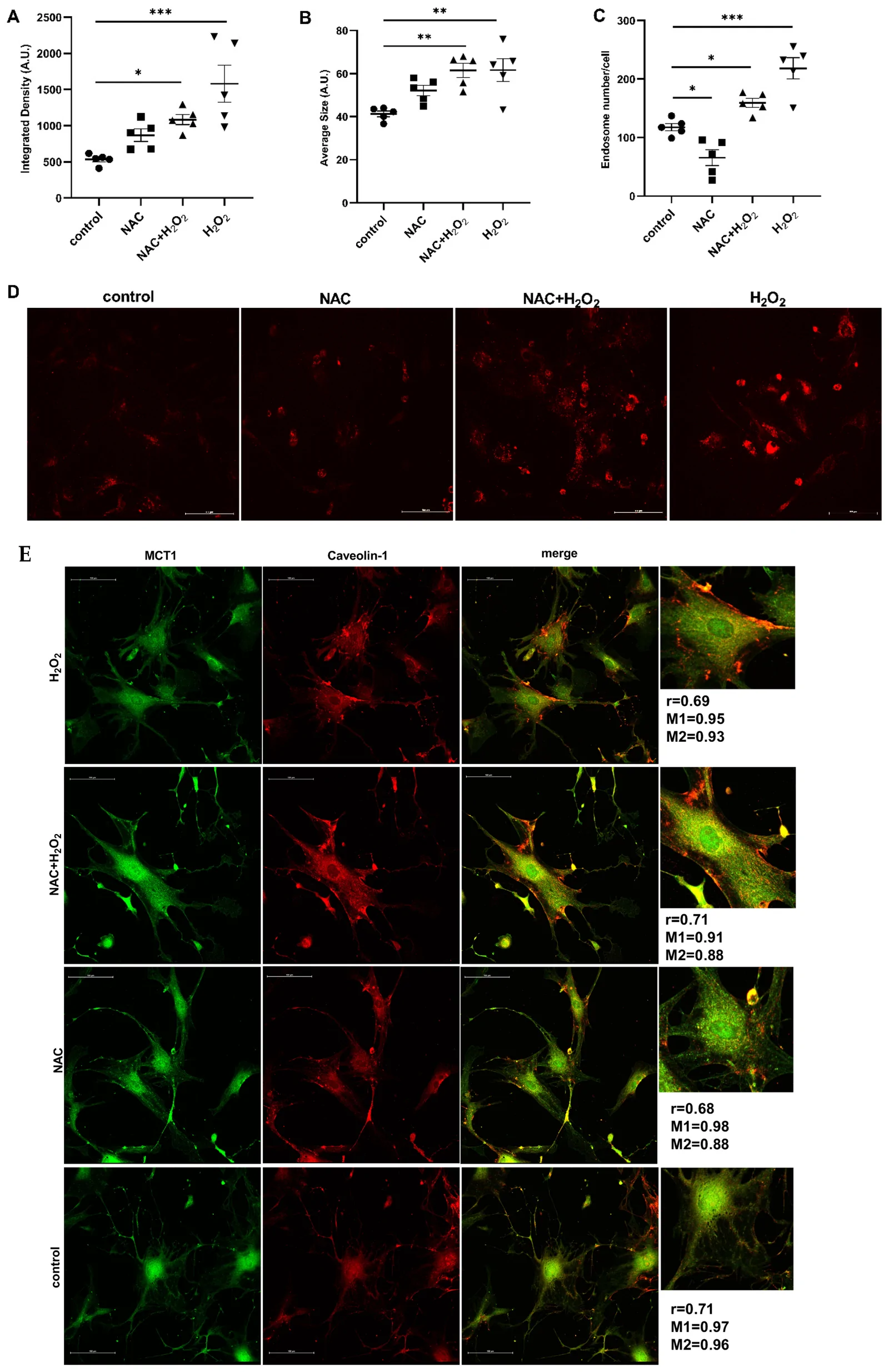
N-acetylcysteine (NAC) treatment partially reverses H_2_O_2_-dependent endocytosis in primary rat podocytes. (**A**) Integrated density and (**B**) average size of live early endosomes. (**C**) Live early endosome number per cell. (**D**) Intracellular endosome distribution. (**E**) Immunofluorescent staining of MCT1 and caveolin-1 in podocytes. r—Pearson’s correlation coefficient; M1, M2—Manders’ overlap coefficients. Cells were treated with: NAC (5 mM for 2 h), H_2_O_2_ (100 µM, 5 min) and NAC + H_2_O_2_. Scale bar is 100 μm, * *p* < 0.05, ** *p* < 0.01, *** *p* < 0.001, one-way ANOVA, *n* = 5.

Considering the pleiotropic and non-specific cellular effects of H_2_O_2_, its endocytosis-stimulating activity may also be associated with the increase in oxidative stress. To prevent the oxidative stress response induced by H_2_O_2_, podocytes were preincubated with NAC, which partially reversed H_2_O_2_-induced endocytosis (Figure 6A–E). NAC slightly and non-statistically increased density and average size of endosomes (Figure 6A,B), but significantly decreased the endosome number per cell by 44% (*p* = 0.03) (Figure 6C). Pre-treatment with NAC mitigated the stimulating effect of H_2_O_2_ on podocyte endocytosis, specifically on endosome density and their number per cell (Figure 6A,C). Although NAC alone did not change the distribution of early endosomes within the cells, H_2_O_2_ and NAC + H_2_O_2_ treatments triggered focal accumulation of early endosomes near the cell membrane (Figure 6D). Moreover, the immunofluorescent staining of caveolin-1 revealed that both in H_2_O_2_ and NAC + H_2_O_2_ conditions its fluorescent intensity was markedly increased near the cell membrane compared to control and NAC alone (Figure 6E). MCT1 and caveolin-1 colocalization was most pronounced in the perinuclear region across all experimental conditions (Figure 6E) and no statistically significant changes were observed in the colocalization between MCT1 and caveolin-1 (Figure S4). These results suggested that the increased endocytosis in podocytes following H_2_O_2_ treatment is not solely a consequence of oxidative stress, but may also reflect the signaling function of H_2_O_2_.

### 2.5. GPR81 as an Upstream Regulator of Cellular Distribution of MCT1

**Figure 7:**
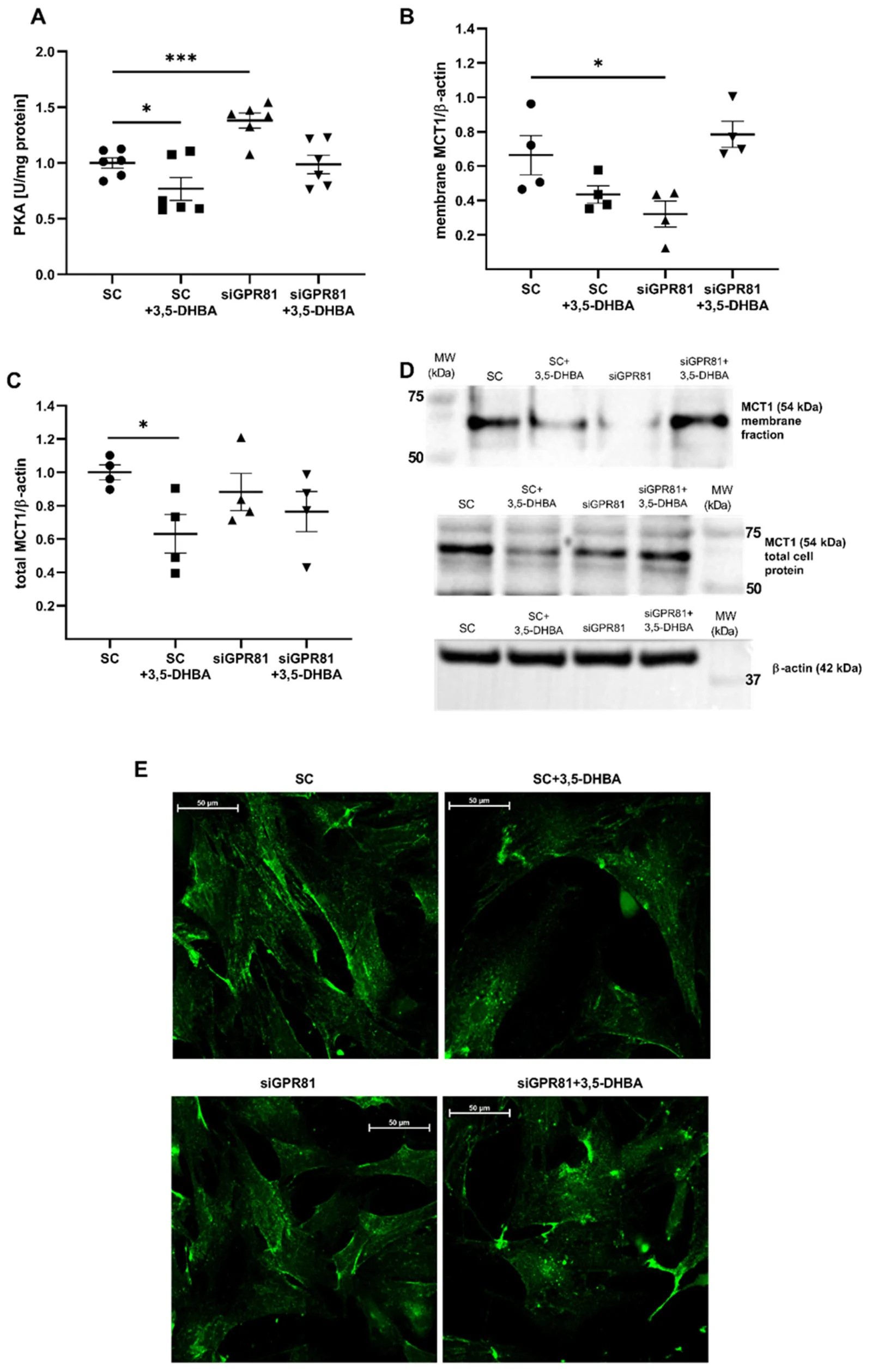
GPR81 signaling influences MCT1 protein expression and translocation from cellular membrane. (**A**) PKA activity (U/mg of protein). (**B**) Membrane content of MCT1 and (**C**) total amount of MCT1 protein. (**D**) Immunoblotting membranes showing MCT1 expression in biotinylated (membrane) and total protein fractions. (**E**) Immunofluorescent staining of MCT1 in podocytes. Podocytes were transfected with scramble siRNA or GPR81 siRNA and treated with 3,5-DHBA (100 µM, 2 h). Cells were visualized using re-scan confocal microscopy (Nikon Eclipse Ti confocal microscope, Nikon Corporation, Tokyo, Japan). Scale bar is 50 µm. * *p* < 0.05, *** *p* < 0.001, one-way ANOVA, Sidak’s multiple comparisons test, *n* = 4–6. SC—scramble RNA, siGPR81—GPR81 silencing, 3,5-DHBA—3,5-dihydroxybenzoic acid.

PKA regulates MCT1 internalization and its kinase activity strongly depends on intracellular cAMP levels. Notably, both cAMP signaling and PKA activity are regulated at least in part by GPR81. Therefore, we investigated whether GPR81-dependent activation of PKA might be an upstream mechanism of MCT1 regulation in podocytes. The GPR81 activator 3,5-DHBA (100 µM, 2 h) and gene silencing with GPR81 siRNA were used to modulate GPR81 activity. The mRNA and protein expression of GPR81 were reduced by 41% and 27%, respectively (Supplementary Figure S5). As expected, PKA activity was reduced (by 23%) in podocytes treated with 3,5-DHBA, and elevated (by 40%) in GPR81-silenced cells. 3,5-DHBA had no effect on PKA activity in podocytes transfected with GPR81 siRNA (Figure 7A).

Analyses of MCT1 expression under these conditions revealed that GPR81 silencing significantly decreased the amount of MCT1 in the cell membrane (by 48%; 0.322 ± 0.0755 vs. 0.644 ± 0.114; *p* = 0.012) (Figure 7B,D). Unexpectedly, stimulation of GRP81 activity also resulted in reduced amount of membrane MCT1, but not in GPR81-silenced podocytes, where 3,5-DHBA restored MCT1 expression to the control levels both in membrane fractions and in total cell lysates (Figure 7C,D). These results were confirmed by fluorescent immunostaining of MCT1 in podocytes treated with 3,5-DHBA or GPR81 siRNA (Figure 7E). Both experimental conditions induced alterations of MCT1 localization, which appeared as focal depositions of MCT1 in central areas of the cells and a lower amount of MCT1 in the cell membrane (Figure 7D).

### 2.6. Alterations of GPR81 Signaling Stimulate Cellular Endocytosis

**Figure 8:**
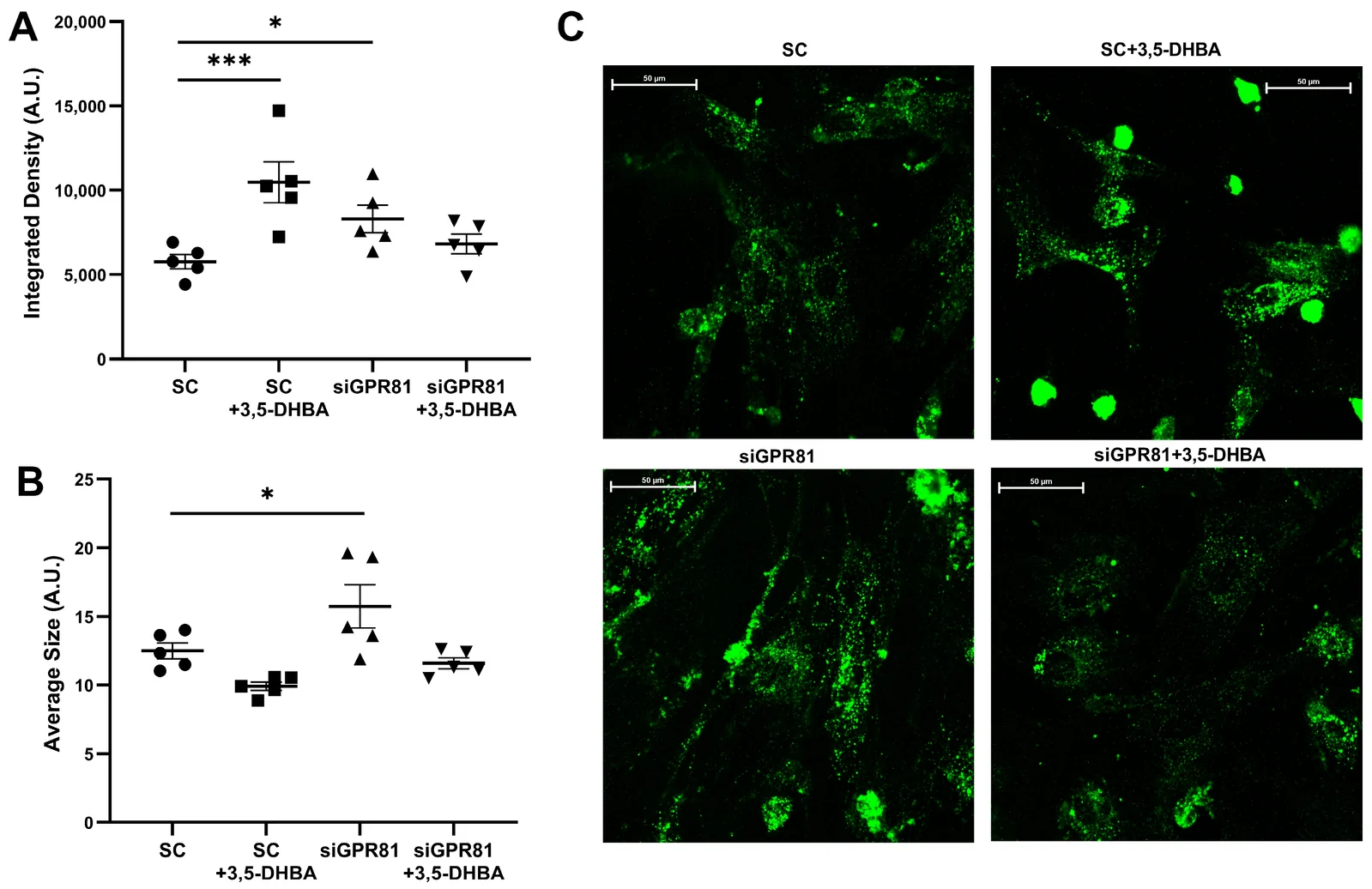
Podocyte endocytosis is regulated by GPR81 signaling. (**A**) Integrated density and (**B**) average size of live early endosomes. (**C**) Distribution of live early endosomes in rat podocytes. Cells were transfected with scramble siRNA or GPR81 siRNA and treated with 3,5-DHBA (100 µM, 2 h). Scale bar is 50 µm, * *p* < 0.05, *** *p* < 0.001, one-way ANOVA, Sidak’s multiple comparisons test, *n* = 5. SC—scramble RNA, siGPR81—GPR81 silencing, 3,5-DHBA—3,5-dihydroxybenzoic acid.

The role of GPCR signaling in regulating the endocytic pathway and enhancing cellular signaling is well established [29]. Previous analyses revealed that MCT1 internalization is dependent on GPR81 signaling. To further explore this relationship, live early endosomes were fluorescently stained and quantified in podocytes following GPR81 activation and inhibition (Figure 8). After 3,5-DHBA exposure and GPR81 silencing, the endosomal density increased by 81% (*p* = 0.0009) and by 44% (*p* = 0.04), respectively (Figure 8A). The average size of endosomes was elevated by 25% after GPR81 downregulation (*p* = 0.02) but no statistical changes were observed after 3,5-DHBA exposure (Figure 8B). Similarly to MCT1 levels, 3,5-DHBA treatment of GPR81-silenced podocytes restored endosomes density and size to control levels (Figure 8A,B). Both experimental treatments increased the fluorescence intensity associated with endosomal vesicles in podocytes (Figure 8C).

## 3. Discussion

MCT1 is abundant in almost all tissues, including the kidneys [30], positioning it as the main transporter among all MCT isoforms. In our previous study, the presence of MCT1 was detected in primary rat podocytes, and changes in the amount of this protein were observed during lactate supplementation and glucose deprivation [7]. However, the exact mechanisms of the physiological regulation of MCT1 have not yet been fully elucidated. In the present study, the mechanism of PKA-mediated MCT1 internalization in primary rat podocytes was revealed. Surprisingly, both PKA activation and inhibition enhanced MCT1 incorporation from the plasma membrane, and co-treatment with H_2_O_2_ and H89 did not exert an additive effect. Despite comparable amounts of MCT1 in the plasma membrane fractions across conditions, qualitative differences emerged in MCT1 distribution and its colocalization with endosomal markers near the cell membrane. In particular, PKA inhibition induced a peripheral redistribution of MCT1 with caveolin-1 and Rab5a. Notably, the overall activity of early endosomes was significantly altered only upon PKA activation—both H_2_O_2_ and PKA activator increased fluorescence intensity and size of early endosomes. Experiments with endocytosis inhibitor (MβCD) showed decreased activity of early endosomes upon combined H_2_O_2_ + MβCD treatment compared to H_2_O_2_ treatment alone, supporting the idea, that H_2_O_2_ activation of PKA resulted in increased endocytosis in podocytes. Additionally, significant downregulation of caveolin-1 in both the membrane and cytosolic fraction was demonstrated—which confirms the effect of MβCD on caveolin-1-dependent pathways. Simultaneously, downregulation of MCT1 cytosolic and membrane (non-significant) fractions upon endocytosis inhibition was revealed, which may suggest a link between podocyte endocytosis regulation and MCT1 protein expression.

Until now, few studies have reported an increase in the membrane trafficking of MCT1 following an increase in the intracellular concentration of cAMP [18] or the activation of adrenergic receptors [31] in other tissues. To our knowledge, PKA-dependent internalization of MCT1 has not been reported in podocytes so far. Paradoxically, we observed decreased MCT1 cell-surface levels under both PKA inhibition and activation. Prior studies show an increased endocytosis of receptor proteins in response to PKA inhibition. For example, Salazar et al. found that the inhibition of basal PKA activity led to the internalization of 40–60% unoccupied, inactive endothelial growth factor receptors and accumulation into early endosomes. This non-classical pathway operated independently of ligand binding, receptor kinase activity, and ubiquitylation and did not trigger receptor degradation [32]. A potential explanation for this phenomenon could be the functional interplay between PKA and AMP-activated protein kinase (AMPK). PKA inhibition leads to AMPK activation [33] and AMPK regulates MCT1 expression and membrane localization [34]. Additionally, PKA can bidirectionally regulate NF-_Κ_B signaling pathway, exerting both inhibitory and activating effects in a context-dependent manner [35,36,37,38], and NF-_Κ_B pharmacological inhibitors reduced MCT1 expression [39]. Taken together, these findings suggest that secondary pathways affected by PKA inhibition may contribute to the reduced MCT1 membrane abundance observed in our study. Further research is required to fully understand these mechanisms.

In podocytes, endocytosis is a fundamental homeostasis process as proper cell surface expression and localization of podocin and nephrin are crucial for maintaining filtration barrier integrity. In this work, elevated endocytic activity was demonstrated after H_2_O_2_ treatment. Experiments utilizing NAC demonstrated that H_2_O_2_ has a wide signaling spectrum and presumably dual mechanism of action in the context of endocytosis. Endocytosis-promoting effect of H_2_O_2_ may originate, at least partially, from direct intracellular H_2_O_2_ signaling rather than being solely a response to oxidative stress.

Both the activation and inhibition of PKA are involved in many pathological conditions. Deb et al. demonstrated that increased cAMP-PKA activity in high glucose concentrations lead to histone hyperacetylation underlying the fibrogenic program in the kidney [40]. Lactate is known to reduce intracellular cAMP levels and suppress PKA activation through a GPR81-dependent mechanism. This dependence in podocytes was demonstrated in our previous study, showing that high glucose concentration increased lactate level and decreased PKA activity in primary rat podocytes [17]. In this work, the impact of GPR81 for MCT1 translocation was investigated. Both GPR81 activation and inhibition led to a reduction in MCT1 at the cell surface, however, only inhibition produced a statistically significant decrease. Notably, GPR81 activation also lowered total cellular MCT1. Moreover, GPR81 activation led to a decrease in the average endosome size and an increase in fluorescence signal, indicating greater endocytic activity. In contrast, GPR81 inhibition resulted in both an increase in endosome size and a higher fluorescence signal, suggesting alterations of vesicular dynamics. Although the precise mechanism of GPR81-induced endocytosis is yet to be fully elucidated, our findings provide the first evidence of an increase in the general rate of endocytosis that is stimulated by GPR81 signaling in rat podocytes.

Under certain conditions, lactate, through GPR81 signaling, can regulate MCT1 expression [41,42], but this has not been previously demonstrated in podocytes. In glioblastoma, lactate and 3,5-DHBA increased the expression of MCT1 [41], and GPR81 knockdown in epithelial MCF7 cells led to decrease in MCT1 expression [42]. These results differ from the present findings, in which both GPR81 inhibition and activation caused the downregulation of total and cell surface MCT1. GPR81-silenced cells treated with 3,5-DHBA exhibited a recovery of membrane MCT1 levels to control values. Given that GPR81 silencing increased PKA activity while GPR81 activation reduced PKA activity and combined treatment did not change the activity of PKA, these results suggest that MCT1 translocation from the cell membrane may be regulated by GPR81 signaling through both PKA-dependent and PKA-independent mechanisms. This is further supported by our findings that both PKA activation and inhibition led to the removal of MCT1 from the cell membrane. Consistent with this, Narumi et al. found that MCT1 expression and lactate transport in skeletal muscle cells were regulated through both PKC- and PKA-mediated pathways, highlighting the complexity of MCT1 regulation [3]. Further studies are required to elucidate this regulatory network.

In our previous studies we have demonstrated that activation of GPR81-PKA signaling resulted in reduced lipolysis and lipid droplet formation [17]. Lipid droplets are enriched in proteins that are involved in membrane trafficking, likely facilitating lipid transport between cellular compartments. Lipid droplet accumulation, caused by the inhibition of lipolysis, is associated with GPR81 activation [43], and enhanced lipid droplets-endosomes contact site formation has been documented [44,45]. In this study, GPR81 signaling affected early endosomes activity, which may be consistent with changes in lipid droplets motility induced by GPR81-controlled lipolysis.

The present study confirms the signaling role of H_2_O_2_ in modulating PKA activity in podocytes and its crosstalk with MCT1 endocytosis. Despite its potential toxicity, H_2_O_2_ conveys redox information essential for cellular homeostasis and metabolic control [46]. Redox–PKA crosstalk is well documented: as a redox-sensitive kinase, PKA can be directly activated by H_2_O_2_ via interprotein disulfide dimer formation [47]. H_2_O_2_-dependent endocytosis was reported in endothelial [48] and tumor cells [49]. H_2_O_2_ signaling seems to be coupled with lactate metabolism. In our previous work, we demonstrated that lactate increased mitochondrial respiratory function in podocytes, indicating a transition toward greater reliance on oxidative phosphorylation for cellular energy generation [8]. The mitochondrial transport chain generates the leakage of electrons that combine with molecular oxygen to create superoxide and hydrogen peroxide [43]. Lactate also has been shown to generate superoxide anions in the electron transport chain of mitochondria [50,51]. Hence, the metabolic state of podocytes that is altered by lactate might contribute to regulation of the intracellular translocation of MCT1, and H_2_O_2_ might trigger this process. Given lactate’s role in upregulating oxidative phosphorylation [8], an increase in ROS, particularly H_2_O_2_, can be expected as a metabolic byproduct [12]. To counteract this rise in mitochondrial ROS production, lactate may contribute to the activation of adaptive cellular mechanisms. Indeed, studies have shown that lactate possesses antioxidant properties; the lactate ion has demonstrated free radical scavenging activity [52]. This oxidative stress adaptation appears to be mediated at least partially by GPR81 activity [53]. While our current data align with the proposed model, future studies are needed to provide the necessary direct evidence to substantiate the role of lactate in modulating oxidative stress and its effect on endocytosis in podocytes. In our previous work, we demonstrated that PKA–GPR81 signaling is downregulated in podocytes under hyperglycemic conditions. Subsequently, we observed a reduction in total MCT1 with a concomitant increase at the cell surface. These changes in the PKA–GPR81 axis were accompanied by actin cytoskeletal reorganization and increased albumin permeability [17].

Together, these findings suggest that MCT membrane translocation may contribute to the regulation of lactate homeostasis in diabetes and correlates with podocyte injury. The present study provides molecular insight into MCT1 endocytic regulation, identifying it as a potential therapeutic target. In our previous study, MCT1 inhibition increased permeability of isolated rat glomeruli and podocyte monolayer to albumin. Combined treatment of MCT1 inhibitor and insulin exacerbated permeability of podocyte monolayer to albumin and podocyte cytoskeleton remodeling, which reveals functional role of MCT1 in glomerular filtration barrier functioning [54].

Dysfunction of the endocytic machinery is implicated in the development of proteinuria and in glomerular and tubular injury in kidney diseases associated with hypertension and diabetes. Notably, several studies show that PKC-α mediates the endocytosis of nephrin [55] and TGF-β receptor I [56] in podocytes, thereby promoting podocyte and glomerular injury; under diabetic conditions, PKC also triggers nephrin [57] and EGFR [58] endocytosis, further exacerbating renal damage. Thus, modulating downstream pathways governed by kinases such as PKA and PKC may be critical for preventing podocyte injury. A limitation of the current study is that the scope did not include a detailed analysis of the endocytosis process and direct evidence that the reduced levels of MCT1 in the cell membrane are caused by endocytosis. The kinetics of functional transport of lactate under given experimental conditions was also not evaluated. Given that GPR81 activity is not fully reduced, other signaling pathways mediated by GPR81 might influence the magnitude of observed effects. While these findings successfully elucidate the GPR81-PKA effects on MCT1 internalization specific molecular mechanism underlying its endocytosis and how MCT1 internalization affects lactate transport in podocytes are yet to be fully characterized in future studies.

## 4. Materials and Methods

### 4.1. Preparation and Culture of Primary Rat Podocytes

All experimental procedures were performed in accordance with directive 2010/63/EU of the European Parliament and of the Council on the protection of animals used for scientific purposes. All experiments were conducted in compliance with the guidelines of University of Gdańsk (Ethical approval for animal euthanasia: 1/D000/2023) and in accordance with the ARRIVE guidelines. 4–5 weeks old female Wistar rats (100–120 g) were used for podocyte isolation. Rats were housed in standard cages on a 12 h/12 h light/dark cycle with free access to a standard pellet diet (Labofeed B standard, Wytwornia Pasz “Morawski,” Kcynia, Poland) and tap water ad libitum. For each procedure reflecting biological replicates, two rats were anesthetized with ketamine/xylazine (100 mg/mL and 20 mg/mL respectively) prior to kidney isolation to obtain an appropriate number of primary cells for the experiments. Kidneys were extracted with surgical scissors and immediately placed in ice-cold phosphate-buffered saline (PBS) containing 1% penicillin-streptomycin solution (P/S). In the next step, they were finely minced with a scalpel and pressed through a system of three sieves with a progressively smaller pore diameter (250, 125, and 75 μm). Following centrifugation, the isolated renal glomeruli were suspended in RPMI 1640 that was supplemented with 10% fetal bovine serum (FBS), 1% P/S and 11 mM D-glucose (SG). After 6 days, the glomeruli were trypsinized and passed through a 30 µm pore-size sieve [59]. Obtained primary rat podocytes were cultured for 12–20 days in SG for all experiments. Each ‘*n*’ represents an independent biological replicate, corresponding to a single podocyte isolation.

For PKA ATP-competitive inhibition, H89 reagent was used (catalog no. CAS 130964-39-5, Santa Cruz Biotechnology, Dallas, TX, USA) at 10 µM concentration for 1 h [58,60]. For specific inhibition of PKA downstream signaling, a pseudosubstrate peptide, myristoylated PKI 14–22 amide was used (catalog no. 476485, Merck KGaA, Darmstadt, Germany) at 10 µM for 30 min. PKA activation was achieved using 8-bromo-cAMP (8-bromo-adenosine-3′,5′-cyclic monophosphate; catalog no. CAS 76939-46-3, Santa Cruz Biotechnology, Dallas, TX, USA) at 100 µM for 5 min. Hydrogen peroxide solution (catalog no. 95321, Merck KGaA, Darmstadt, Germany) was also used for non-specific modulation of PKA activity (100 µM, 5 min). H_2_O_2_ and 8-bromo-cAMP concentration and time of incubation were selected based on our previous experiments [58].

For GPR81 activation, the GPR81 agonist 3,5-dihydroxybenzoic acid (3,5-DHBA; catalog no. D110000, Sigma Aldrich, Merck KGaA, Darmstadt, Germany) was used at 100 µM concentration for 2 h.

Endocytosis was inhibited by Methyl-β-cyclodextrin (MβCD; catalog no. T4072, TargetMol Inc., Boston, MA, USA) at 5 mM for 1 h, following Sohn et al. work [61].

As an antioxidant agent N-Acetyl-L-cysteine (NAC) was used at 5 mM for 2 h (catalog no. A9165, Merck KGaA, Darmstadt, Germany). Time of podocyte incubation with NAC and its concentration were established according to Montero et al. work [62].

All compounds were dissolved in water or DMSO, so that the final concentration did not exceed 0.5% *v*/*v* of the culture medium.

### 4.2. RNA Isolation, Reverse Transcription, and Polymerase Chain Reaction (PCR)

Total RNA from podocytes was isolated with the RNeasy Mini Kit (Qiagen, Hilden, Germany), which is based on spin column extraction. A NanoDrop UV/Vis Spectrophotometer (Thermo Fisher Scientific, Waltham, MA, USA) was used for RNA purity and quantity assessment. Isolated RNA was transcribed into cDNA and used for real-time quantitative PCR (qPCR) with TaqMan probes and sequence-specific primers to establish levels of gene expression. For quantification of the results, the ΔΔCt method was used, with β-actin as an internal control. The analyses were performed using LightCycler480 (Roche Diagnostics, Rotkreuz, Switzerland). The PCR products were separated on 2.5% agarose gel and detected by the GelDoc-It Imaging System (UVP, Cambridge, UK). Sequences of primers and probes that were used in the experiments are listed in Table 1.

### 4.3. Western Blot

Podocyte cell lysates were prepared in the presence of a lysis buffer (1% Nonidet P-40, 20 mM Tris, 140 mM NaCl, 2 mM Ethylenediaminetetraacetic acid (EDTA), 10% glycerol) and protease inhibitor cocktail (TargetMol Chemicals Inc., Boston, MA, USA). Cells were scraped at 4 °C and centrifuged at 18,000× *g* for 20 min at 4 °C [59]. Sodium dodecyl sulfate-polyacrylamide gel electrophoresis was performed with an equal total amount of protein (20 µg) per sample. Proteins were transferred to polyvinylidene difluoride membranes, followed by incubation with Tris-buffered saline (TBS) (20 mM Tris-HCl pH 7.4, 140 mM NaCl) containing 4% non-fat dry milk for 2 h to block non-specific binding sites. In the next step, immunodetection was performed by incubating the membranes for 12 h at 4 °C with specific primary antibodies diluted in TBS with addition of 5% Bovine Serum Albumin (BSA). Then, the membranes were incubated with horseradish peroxidase-conjugated secondary antibodies diluted 1:3000 in TBS with 5% BSA (Invitrogen, Thermo Fisher Scientific, Waltham, MA, USA). Chemiluminescent detection with luminol-based electrochemiluminescence substrates was performed (Bio-Rad Laboratories, Hercules, CA, USA). All primary antibodies that were used in the experiments are listed in Table 2.

### 4.4. Immunofluorescence and Early Endosome Imaging

For immunofluorescence immunostaining, podocytes were seeded on glass dishes (35 mm) that were coated with type I rat collagen. Cells were fixed with 4% paraformaldehyde and permeabilized with 0.1% Triton-X 100 and then blocked with 3% bovine serum albumin in PBS for 1 h. Next, primary antibodies that were diluted in 3% bovine serum albumin in PBS were added for 12 h. The next day, the cells were washed with PBS three times and incubated with fluorescently conjugated secondary antibodies for 1 h (Alexa, Thermo Fisher Scientific, Waltham, MA, USA). Cells were washed with PBS and H_2_O and coverslips were mounted on the glass slides. Cells were analyzed with a confocal laser scanning microscope (Nikon Ti Eclipse, Nikon Corporation, Tokyo, Japan; 40× objective).

For early endosome imaging, podocytes were washed, and FluoroBrite Dulbecco’s Modified Eagle Medium (DMEM; catalog no. A1896701, Thermo Fisher Scientific, Waltham, MA, USA) was applied. pHrodo Green Dextran (catalog no. P35368, Thermo Fisher Scientific, Waltham, MA, USA) diluted 30 µL per 1 mL of FluoroBrite DMEM was added to living cells and placed for 30 min at 37 °C. Early endosomes were analyzed with a confocal laser scanning microscope (40× objective).

### 4.5. Quantification of Fluorescent Images

Colocalization was evaluated using the Coloc 2 plugin in Fiji 1.54p software (Wayne Rasband and contributors, National Institutes of Health, Bethesda, MD, USA). For each image, single cells were manually defined as regions of interest (ROIs) and Bisection thresholding was applied. The degree of colocalization was quantified using Pearson’s correlation coefficient (*r*) and Manders’ overlap coefficients (M1 and M2).

The average size of early endosomes, number of early endosomes per cell and integrated density were measured with Fiji 1.54p. Each *n* corresponds to an individual image, and for each, the mean fluorescence intensity and mean endosomal size per cell were calculated.

### 4.6. Cell Surface Biotinylation Assay

Podocytes were incubated with PBS that contained 1 mg/mL Sulfo-NHS-SS-biotin (EZ-Link NHS-Biotin, Thermo Fisher Scientific, Waltham, MA, USA) for 30 min. After incubation, the cells were washed three times with cold PBS that contained 100 mM glycine. Lysates were prepared and added to neutravidin beads (Pierce NeutrAvidin UltraLink Resin, Thermo Fisher Scientific, Waltham, MA, USA) at 4 °C for 12 h with rotary spinning. Neutravidin beads were washed with 2× loading buffer, and proteins were denatured for 10 min at 96 °C. The samples were centrifuged, the beads were discarded, and the supernatants were subjected to a standard Western blot procedure.

### 4.7. Separation of the Membrane and Cytosolic Fraction

Podocytes were homogenized in lysis buffer (30 mM Tris, pH 7.5, 10 mM ethylene glycol-bis(β-aminoethyl ether)-N,N,N′,N′-tetraacetic acid (EGTA), 5 mM EDTA, 1 mM dithiothreitol (DTT), 250 mM sucrose) in the presence of protease inhibitor cocktail (TargetMol Chemicals Inc., Boston, MA, USA) for subsequent ultracentrifugation [63]. The lysates were centrifuged at 9500× *g* for 10 min at 4 °C. Next, ultracentrifugation of supernatant was performed at 60,000× *g* for 40 min at 4 °C and supernatant was used as a cytosolic fraction. To obtain membrane fraction, remaining pellet was resuspended in lysis buffer without sucrose and solubilized with 1% Triton X-100. Membrane and cytosolic fractions of caveolin-1 and MCT1 were further examined by Western blot.

### 4.8. Cell Transfection with siRNA

The knockdown of GPR81 gene expression was accomplished through cell transfection with GPR81-specific siRNA (catalog no. 4390815, Thermo Fisher Scientific, Waltham, MA, USA). For the negative control, non-silencing siRNA (catalog no. 4390843, Thermo Fisher Scientific, Waltham, MA, USA) was used. Twelve hours before the experiment, the culture medium was replaced with antibiotic-free RPMI-1640 that was supplemented with 10% FBS. Podocytes were transfected using Lipofectamine 3000 diluted in Opti-MEM (Gibco, Thermo Fisher Scientific, Waltham, MA, USA) according to the manufacturer’s instructions. Transfection reagent and siRNA were incubated with Opti-MEM for 5 min and then combined for 15 min of incubation. Complexes of siRNA and transfection reagent were added to the cell cultures and incubated at 37 °C. After 7 h, medium with a two-fold higher concentration of FBS and antibiotics was added to podocytes (1:1 *v*/*v*) for the next 24 h. Gene silencing was examined by qPCR and Western blot.

### 4.9. Quantification of PKA Activity

Protein kinase A activity was measured in the cell lysate using the commercially available kit PKA Colorimetric Activity Kit (catalog no. EIAPKA, Invitrogen, Thermo Fisher Scientific, Waltham, MA, USA) according to the manufacturer’s instructions.

### 4.10. Statistical Analysis

GraphPad Prism 8 software (Boston, MA, USA) was used for all statistical analyses. To determine whether parametric or nonparametric tests should be conducted, the Shapiro–Wilk test was performed. The data were analyzed using one-way analysis of variance (ANOVA). The results are expressed as the mean ± SEM. Values of *p* < 0.05 were considered statistically significant.

## 5. Conclusions

**Figure 9:**
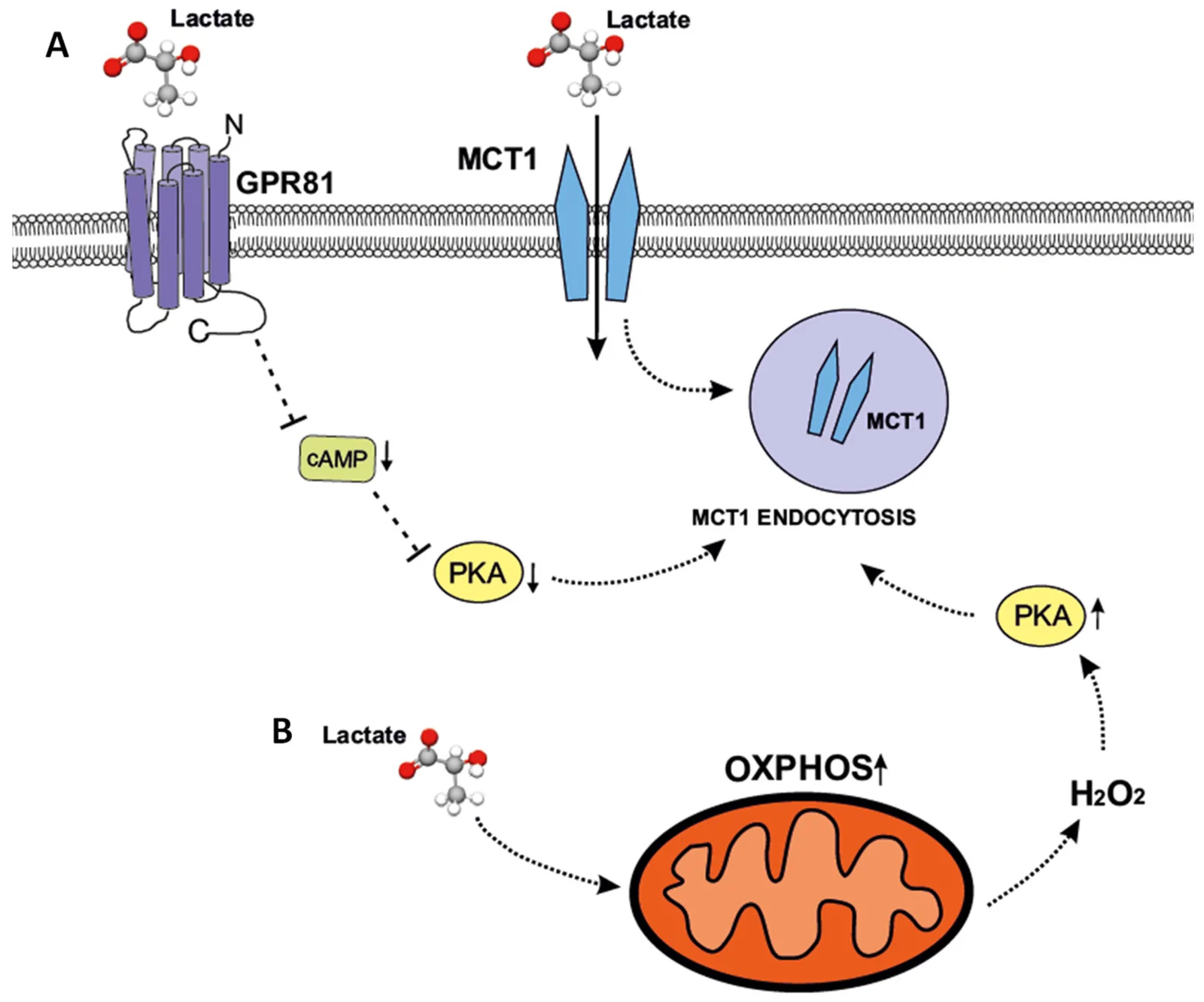
Proposed mechanism of MCT1 regulation in podocytes. Both PKA activation and inhibition result in enhanced MCT1 endocytosis. Presence of extracellular lactate could decrease MCT1 membrane content through GPR81 activation (**A**). Intracellular lactate may increase production of reactive oxygen species (e.g., H_2_O_2_) through an upregulation of oxidative phosphorylation (OXPHOS), which subsequently promotes endocytosis of MCT1 (**B**). Dashed arrows represent proposed mechanism. T-bar arrows indicate inhibitory effects.

In summary, the relationship between GPR81 signaling and MCT1 endocytosis is mediated at least partially by PKA regulation via both cAMP-dependent and -independent mechanisms. PKA as a key regulator (whether inhibited or activated) can influence MCT1 cell surface expression. These findings suggest that lactate may regulate the surface availability of MCT1 through both activation of GPR81 signaling and its role as a redox molecule, resulting from an increase in oxidative metabolism and subsequent elevation of H_2_O_2_ production (Figure 9). This ensures metabolic flexibility and homeostasis in response to changing environmental conditions. Overall, our study provides evidence of the involvement of the GPR81–PKA pathway in regulating endosomal dynamics and MCT1 trafficking and underscores the complexity of this process, which involves multiple downstream effectors.

## Figures and Tables

**Table 1:** Sequences of primers and probes used in the experiments.

Protein Name	Gene Name	Accession No. for mRNA Sequence	Primer Sequence	Probe Sequence	Product Length (bp)
GPR81	*Hcar1*	NC_051347.1	Forward: 5’TTTACTCATCCTGGCCTTCC3’ Reverse: 3’AGAAACCACACAGGGCTAGG5’	CTTGGAGC	149
Β-actin	*Actb*	NM_031144.3	Forward: 5’AGCCCCTCTGAACCCTA3’ Reverse: 3’GGGGTGTTGAAGGTCTCAAA5’	CGTGAAAAGATG	72

**Table 2:** Primary antibodies used in the experiments.

Antibody	Dilution	Source
MCT1	1:1000 (Western blot) 1:400 (Western blot) 1:20 (Immunofluorescence)	Abclonal; catalog no. A3013 Merck; catalog no. AB1286-I SantaCruz; catalog no. sc-50325
Caveolin 1	1:50 (Immunofluorescence)	SantaCruz; catalog no. sc-53564
Rab5a	1:50 (Immunofluorescence)	SantaCruz; catalog no. sc-166622
β-Actin	1:10,000 (Western blot)	Sigma-Aldrich; catalog no. A5441

## Data Availability

The data that support the findings of this study are available from the corresponding author, upon reasonable request.

## Supplementary Materials

The following supporting information can be downloaded at: https://www.mdpi.com/article/10.3390/ijms27177563/s1.

## Abbreviations

The following abbreviations are used in this manuscript:

NAC	N-Acetyl-L-cysteine
3,5-DHBA	3,5-dihydroxybenzoic acid
MβCD	Methyl-β-cyclodextrin
