# Supplementary material for: GPR81 Regulates MCT1 Membrane Translocation Through a PKA-Dependent Signaling Pathway in Rat Podocytes
Source: Int J Mol Sci. 2026 Aug 24;27(17):7563. doi: 10.3390/ijms27177563 (PMC13566832; doi:10.3390/ijms27177563)
Supplement: Supplementary file 1 [file ijms-27-07563-s001.zip › ijms-4461758-supplementary.pdf]

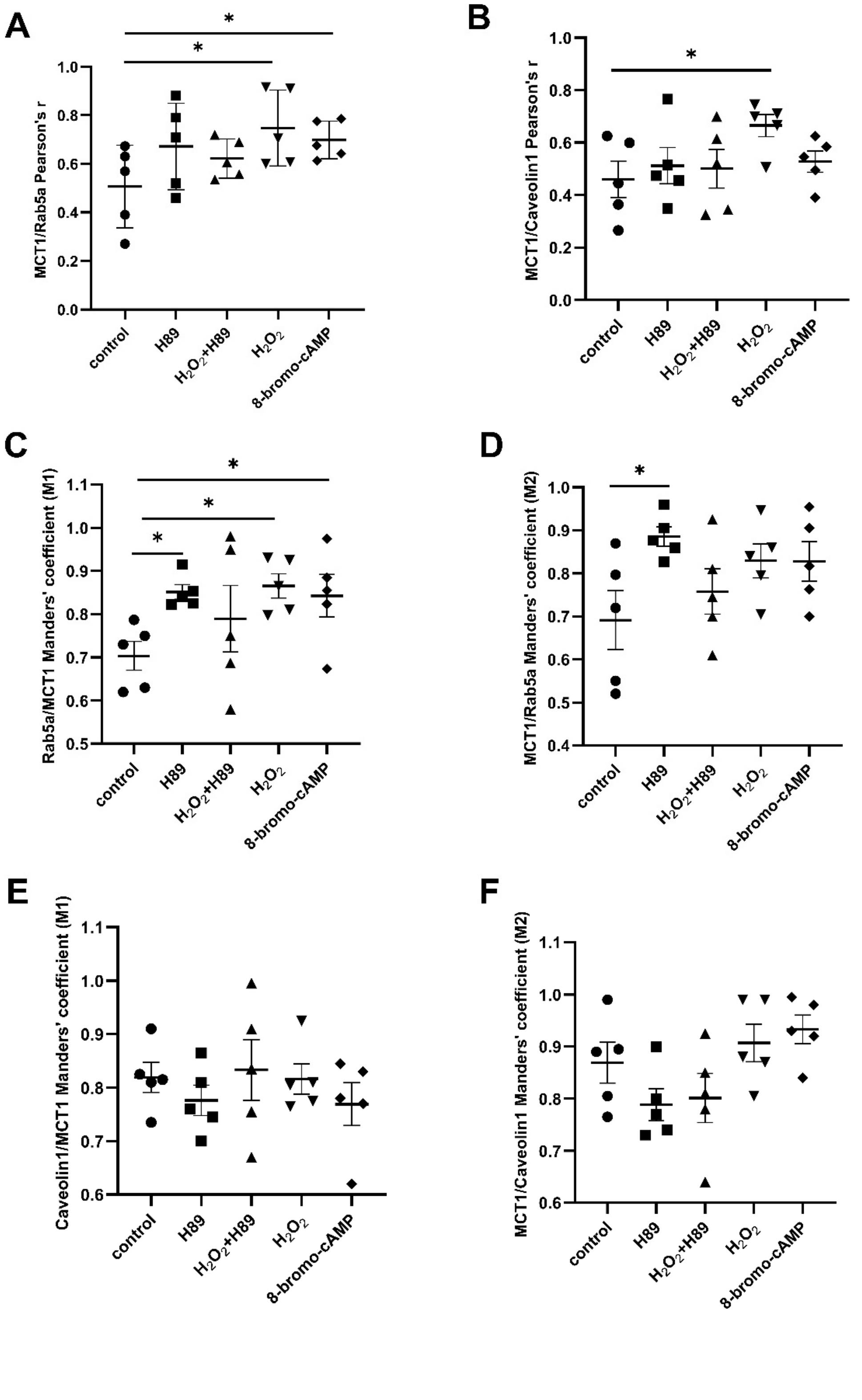

Supplementary Figure S1. Quantitative colocalization analysis of MCT1/Rab5a and MCT1/Caveolin1 in primary rat podocytes supporting Figure 2. Pearson's correlation coefficient (r) of MCT1 and Rab5a (A) and MCT1 and Caveolin 1 (B) indicating overall spatial correlation. Manders' overlap coefficients M_1 (fraction of Rab5a overlapping with MCT1) (C) and M_2 (fraction of MCT1 overlapping with Rab5a) (D). Manders' overlap coefficients M_1 (fraction of Caveolin 1 overlapping with MCT1) (E) and M_2 (fraction of MCT1 overlapping with Caveolin 1) (F). Cells were treated with: 8-bromo-cAMP (100 µM, 5 min), H_2O_2 (100 µM, 5 min), H89 (10 µM, 1 h), or H_2O_2+H89. *$p < 0.05$, **$p < 0.01$, one-way ANOVA, n=5

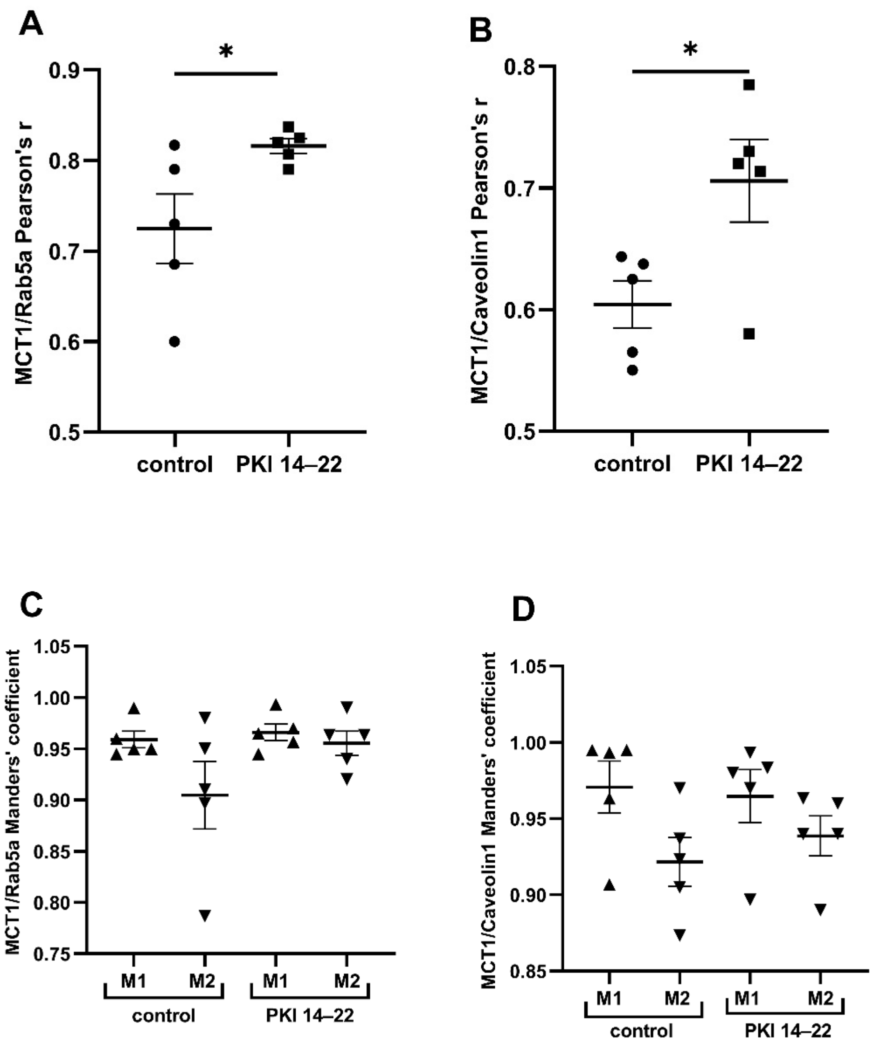

Supplementary Figure S2. Quantitative colocalization analysis of MCT1/Rab5a and MCT1/Caveolin1 in primary rat podocytes supporting Figure 3. Pearson's correlation coefficient (r) of MCT1 and Rab5a (A) and MCT1 and Caveolin 1 (B) indicating overall spatial correlation. Manders' overlap coefficients M_1 (fraction of MCT1 overlapping with Rab5a) (C) and M_2 (fraction of Rab5a overlapping with MCT1) (D). Manders' overlap coefficients M_1 (fraction of MCT1 overlapping with Caveolin 1) (E) and M_2 (fraction of Caveolin 1 overlapping with MCT1) (F). Cells were treated with PKI 14-22 (10 μM) for 30 minutes $*p < 0.05$ *t*-test, one-way ANOVA, n=5

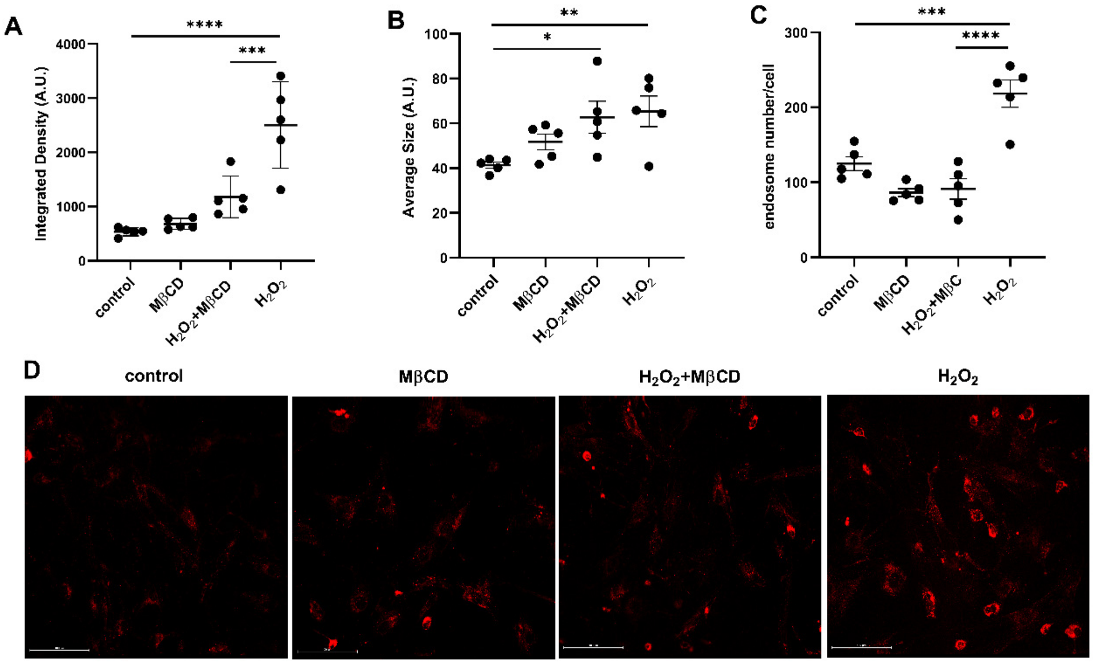

Supplementary Figure S3. Endocytosis inhibitor (MβCD) decreased H_2O_2-dependent cellular endocytosis. (A) Integrated density and (B) average size and (C) endosome number per cell of live early endosomes and (D) their distribution in rat podocytes. Cells were treated with: MβCD (5 Mm, 1 h), H_2O_2 (100 µM, 5 min) and MβCD+ H_2O_2. Scale bar, 100 µm, **p* < 0.05, ***p* < 0.01, ****p* < 0.001, *****p* ≤ 0.0001, one-way ANOVA, *n*=5

To examine whether the MβCD impacts caveolin-1-dependent endocytosis, its effect of on H_2O_2-activated cellular endocytosis was tested (Fig. S3). The integrated density (Fig. S3 A), average size (Fig. 5B) and number of endosomes per cell (Fig. S3 C) were calculated and as it was established earlier, H_2O_2 increased all mentioned endocytosis parameters: integrated density by 368% ($p < 0.0001$), average size by 58% ($p = 0.005$) and endosome number per cell by 74% ($p = 0.0002$) compared to control. MβCD did not affect the basal endocytosis but preincubation with MβCD in pair with H_2O_2 treatment resulted in decreased endocytosis parameters (integrated density and endosome number per cell) compared to H_2O_2 alone. MβCD and MβCD+H_2O_2 treatment did not change localization of early endosomes in podocytes compared to control. Consistent with the earlier experiment, H_2O_2 treatment caused local endosome accumulation near the cell membrane

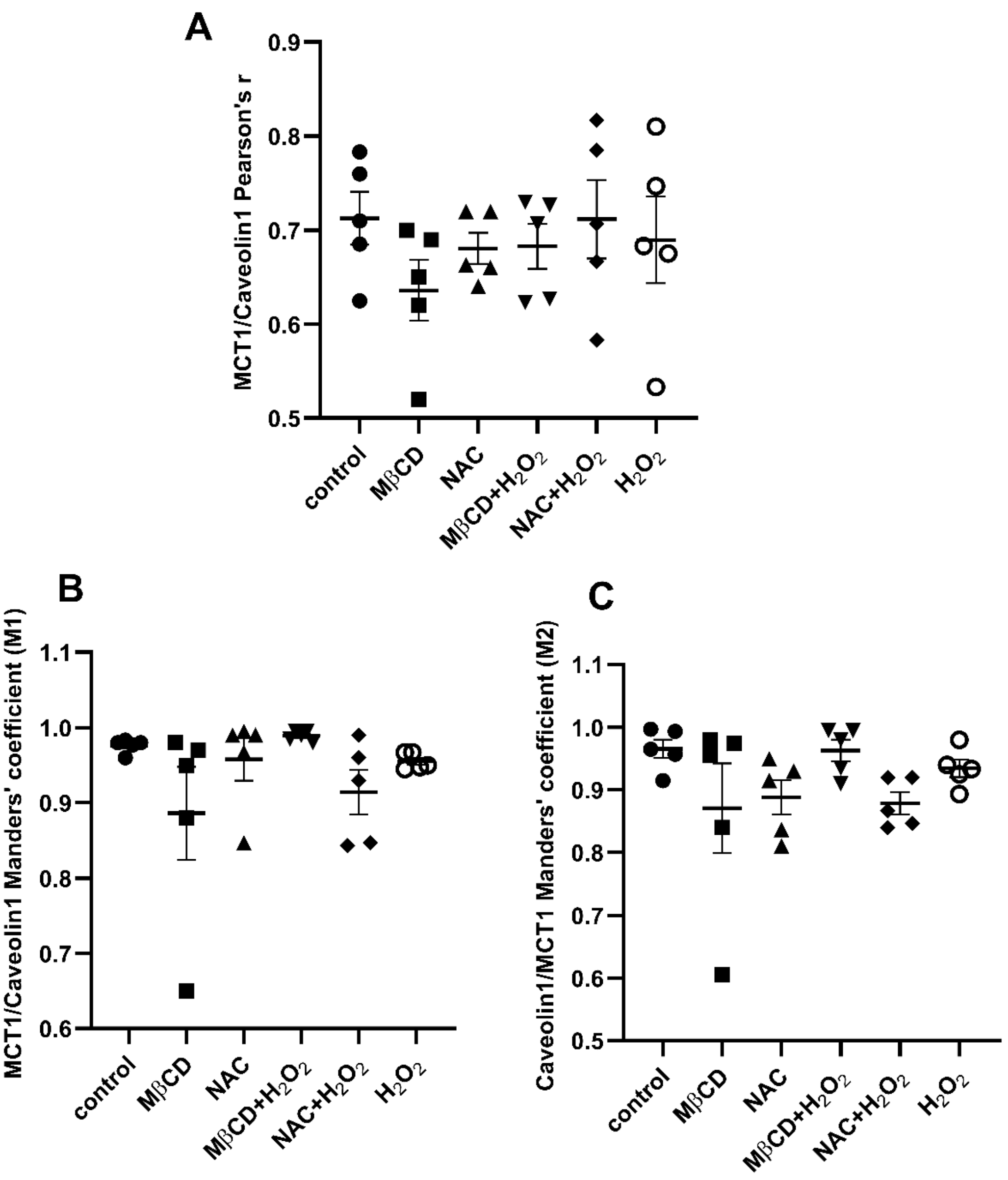

Supplementary Figure S4. Quantitative colocalization analysis of MCT1 and Caveolin1 in primary rat podocytes supporting Figure 5 and 6. Pearson's correlation coefficient (r) of MCT1 and Caveolin 1 (A) indicating overall spatial correlation. Manders' overlap coefficients M_1 (fraction of MCT1 overlapping with Caveolin 1) (B) and M_2 (fraction of Caveolin 1 overlapping with MCT1) (C). Cells were treated with: MβCD (5 Mm, 1 h), NAC (5 mM, 2 h), H_2O_2 (100 µM, 5 min), MβCD+ H_2O_2 and NAC+H_2O_2, *n*=5.

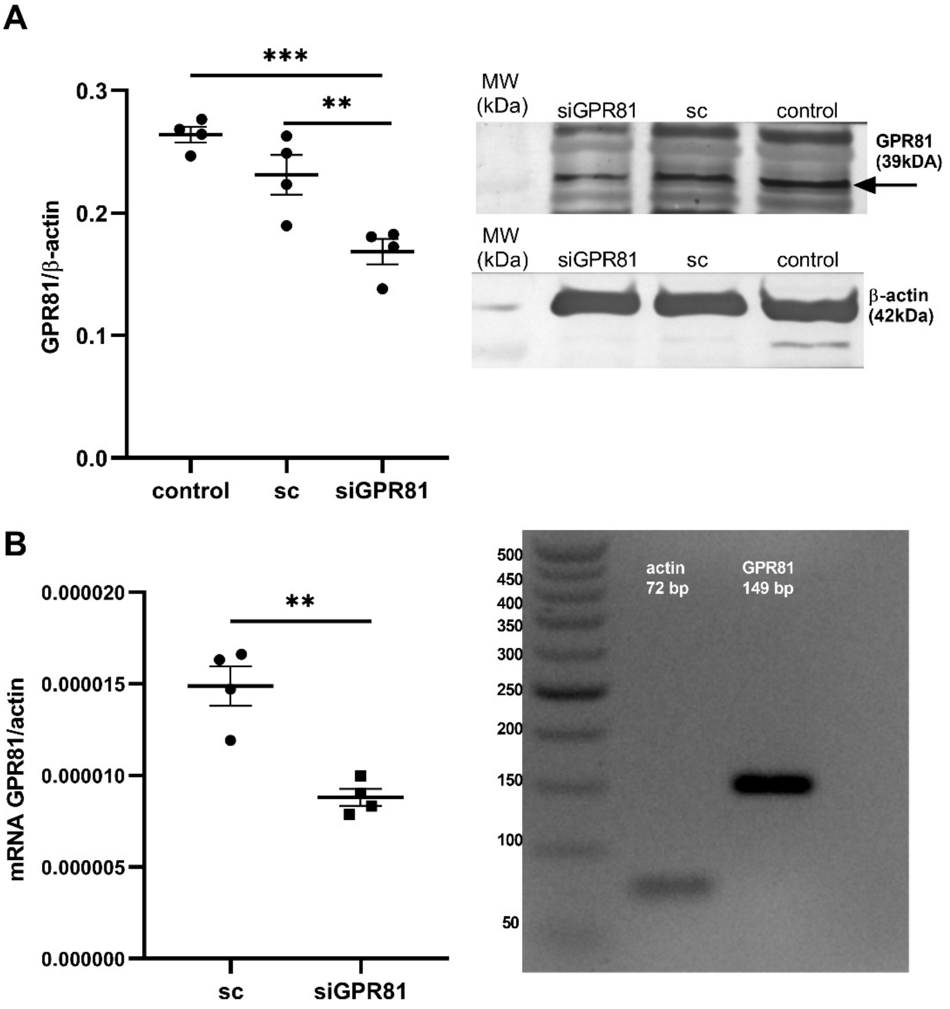

Supplementary Figure S5. GPR81 gene silencing. Effects of GPR81 activation on selected parameters of lipolysis pathway. (A) GPR81 protein expression and (B) mRNA expression visualized by western blot and qPCR. Cells were transfected with scramble siRNA, GPR81 siRNA and treated with 3,5-DHBA (100 µM, 2h) **p<0.01, ***p<0.001, One-way ANOVA, and t-test, n=4

The efficiency of GPR81 gene silencing was evaluated at both mRNA and protein level (Fig. S2). 27% of GPR81 protein expression was decreased compared to scrambled siRNA (p=0.0083) and by 36% compared to control (p=0.0005) (Fig. S5 A). GPR81 mRNA expression was decreased by 41% compared do scrambled siRNA (p=0.0020) (Fig. S5 B).
